# Effect of high-intensity interval training in patients with chronic hepatitis B and hepatic steatosis: A randomised controlled trial

**DOI:** 10.1371/journal.pone.0351547

**Published:** 2026-06-23

**Authors:** Sofie Jespersen, Asmita Fritt-Rasmussen, Cody Garett Durrer, Eva Fallentin, Ellen Sloth Andersen, Peter Thielsen, Thomas Bandholm, Sten Madsbad, Bente Klarlund Pedersen, Christian Ritz, Nina Weis, Rikke Krogh-Madsen

**Affiliations:** 1 Center for Physical Activity Research, Copenhagen University Hospital, Rigshospitalet, Copenhagen, Denmark; 2 Department of Infectious Diseases, Copenhagen University Hospital, Hvidovre, Denmark; 3 Department of Radiology, Copenhagen University Hospital, Rigshospitalet, Copenhagen, Denmark; 4 Department of Medicine, Unit of Infectious Diseases, Copenhagen University Hospitals Herlev and Gentofte, Herlev and Gentofte, Denmark; 5 Department of Gastroenterology and Hepatology, Copenhagen University Hospital Herlev, Herlev, Denmark; 6 Department of Physical and Occupational Therapy, Copenhagen University Hospital, Hvidovre, Denmark; 7 Department of Orthopedic Surgery and Department of Clinical Research, Copenhagen University Hospital, Hvidovre, Denmark; 8 Department of Endocrinology, Copenhagen University Hospital, Hvidovre, Denmark; 9 Department of Clinical Medicine, Faculty of Health and Medical Sciences, University of Copenhagen, Copenhagen, Denmark; 10 National Institute of Public Health, University of Southern Denmark, Odense, Denmark; Instituto Nacional de Ciencias Medicas y Nutricion Salvador Zubiran, MEXICO

## Abstract

**Background & aims:**

Having both chronic hepatitis B and hepatic steatosis increases the risk of liver-related morbidity and mortality. We aimed to investigate if high-intensity interval training could decrease the liver fat-fraction and improve liver status, body composition, lipid- and glucose metabolism, and blood pressure in patients with chronic hepatitis B and hepatic steatosis.

**Methods:**

In a randomised, controlled trial, patients with chronic hepatitis B and hepatic steatosis were randomised 1:1 to high-intensity interval training over 12 weeks or no intervention. The primary outcome was reduction in liver fat-fraction (≥ 2.8%), assessed by magnetic resonance imaging. Secondary outcomes were body composition, indices of glucose metabolism, blood lipids, blood pressure, alanine aminotransferase, physical fitness assessed by maximal oxygen consumption (VO_2_max), and self-assessed health. The trial was discontinued before reaching the planned sample size due to slow recruitment.

**Results:**

Nineteen patients were included, and 14 completed the trial (seven per group). Changes in liver fat-fraction showed a between-group difference of −2.03% [95% CI: −5.5 to 1.4; p = 0.22]. Exercising patients improved VO_2_max by 5.6 mL/kg/min [1.7 to 9.5; p < 0.05] and emotional well-being by 18.07 points [6.6 to 29.6; p < 0.05]. The exercise intervention did not affect total body fat −0.35% [−2.8 to 2.1] or lean body mass 0.6 kg [−0.7 to 1.9]. Changes in glucose tolerance, insulin secretion, Matsuda index −0.89 [−2.5 to 0.5; p = 0.18], cholesterol 0.29 mmol/L [−0.3 to 0.9; p = 0.32] and systolic blood pressure 6.36 mmHg [−6.4 to 19.1; p = 0.29] did not differ significantly between groups.

**Conclusions:**

We did not observe a significant effect of high-intensity interval training on liver fat-fraction in patients with chronic hepatitis B and hepatic steatosis. Due to premature discontinuation and the resulting limited sample size, the study was underpowered, and the findings should be interpreted as exploratory. Nevertheless, the intervention improved physical fitness and emotional well-being.

## Introduction

Chronic hepatitis B (CHB) caused by hepatitis B virus (HBV) poses a significant risk for progression to cirrhosis and liver cancer [1]. Currently, there is no cure to eradicate HBV, but antiviral medication can effectively reduce HBV-related complications [2]. There is a global increase in obesity and cardiometabolic-related diseases, which also affects patients with CHB; these patients have been shown to have higher body mass index (BMI) and poorer physical fitness compared to persons without CHB [3,4]. Low physical activity and obesity increase the risk of metabolic disease and, thereby, non-alcoholic fatty liver disease (NAFLD) [5]. Prevalence of hepatic steatosis in CHB is, similar to the general population, about 35% [6] and carries with it an increased risk of cirrhosis and liver cancer compared to CHB patients without hepatic steatosis [7]. Hepatic steatosis in patients with CHB may differ from steatosis of metabolic origin in terms of underlying pathophysiology. In CHB, viral factors and host–virus interactions may influence lipid metabolism and disease progression, raising uncertainty as to whether treatment strategies established for NAFLD can be directly applied to this population.

The European Association for the Study of the Liver (EASL) guidelines provide no guidance on how to manage patients with both CHB and hepatic steatosis [8]. Previous similar exercise intervention studies in patients with NAFLD (without CHB) have shown decreased liver fat-fraction and improved peripheral insulin resistance and liver fatty acid metabolism [9–11]. Therefore, because patients with CHB have been excluded from these studies, evidence for similar benefits in this group is lacking.

In patients with CHB and hepatic steatosis, this study primarily aimed to investigate whether 12 weeks of high-intensity interval training (HIIT) could decrease the liver fat-fraction. Secondly, to investigate if HIIT could reduce obesity and improve lipid- and glucose metabolism, liver status (by alanine aminotransferase (ALT), fibrosis index-4 (FIB-4) and transient elastography (TE)-score), body composition, and blood pressure.

## Materials and methods

### Study design

This is the primary trial report for ClinicalTrial.gov registration number NCT05265026: A randomized, controlled, superiority trial comparing 12 weeks of HIIT versus no intervention in patients with CHB and hepatic steatosis.

### Participants and eligibility

Patients with CHB from the Department of Infectious Diseases, Copenhagen University Hospital, Hvidovre, Denmark, were included during regular outpatient visits. The inclusion criteria were: Positive hepatitis B surface antigen (HBsAg) for >6 months, positive HBV-DNA, age ≥ 30 years, hepatic steatosis diagnosed by controlled attenuation parameter (CAP) >250 dB/m or by ultrasound scans. The criteria of exclusion were: human immunodeficiency virus, hepatitis C virus, or hepatitis D virus co-infection, primary biliary cholangitis, Wilson’s disease, autoimmune hepatitis, hepatocellular carcinoma, antiviral medication use, steatogenic medication use, contraindications for MRI scan, average daily alcohol intake >30 g for men and >20 g for women, coronary artery disease contraindicating HIIT, inability to understand and read written information for participant’s written consent and pregnancy [12].

### Intervention

The intervention is previously described in detail [12]. In brief, it consisted of 40 minutes of supervised HIIT, and was conducted three times per week, where four intervals lasting four minutes required a heart rate > 85% of heart rate maximum (HRmax); in-between intervals were 3 minutes, with active recovery at 50–70% of HRmax. The HRmax was derived from the maximal oxygen consumption (VO_2_max) test at the baseline visit [12]. Both study groups were orally instructed not to make life changes, such as dietary or exercise, during the trial. The supervision of each exercise session was performed by the Center of Physical Activity Research personnel including students of sports science, physiotherapy students and medical students. Adherence was assessed and recorded by the supervising trainers during each session using exercise logs and heart rate monitoring. For sessions with missing heart rate data, exercise duration was recorded, and the participant’s average training intensity from other sessions was used to estimate intensity.

### Outcomes

#### Liver fat-fraction.

NAFLD is defined by hepatic steatosis exceeding 5% in the hepatocytes [13] and the absence of excessive alcohol intake but excludes a diagnosis of viral hepatitis. Recently, a new terminology of metabolic dysfunction-associated steatotic liver disease (MASLD) [14] allows the inclusion of CHB patients who fulfil the requirements of hepatic steatosis and cardiometabolic abnormalities. However, this study was designed before the implementation of MASLD, and the cardiometabolic requirements (increased BMI, type 2 diabetes, hypertension, or dyslipidaemia) were not included in the inclusion criteria; hence, this study will refer to the patients as having hepatic steatosis. Liver fat-fraction, the primary outcome, was assessed by MRI proton density fat-fraction (MRI-PDFF) [12] after an overnight fast. MRI-PDFF is a well-validated, non-invasive method for quantifying liver fat, demonstrating high diagnostic accuracy and reproducibility. Meta-analytic data show excellent performance across steatosis grades, with areas under the curve (AUC) values ranging from 0.91 to 0.97 and high sensitivity and specificity, supporting its robustness for both detection and grading of hepatic steatosis [15]. The scans were analysed using PhiIlips Intellispace Portal Client v12.1.8.289.

#### Liver parameters.

Transient elastrography (TE) was performed using Vibration-Controlled Transient Elastography (Fibroscan 502 Touch) to assess liver stiffness (via TE-score) and hepatic fat accumulation by CAP. TE performed prior to inclusion was not consistently conducted under fasting conditions, however intended, as these assessments were carried out during routine outpatient visits where fasting could not be systematically ensured. All patients fasted for 3 hours before TE follow-up. Blood for analysis of ALT, FIB-4 score, viral load, and hepatitis B e antigen was analysed using standard procedures at the Department of Clinical Biochemistry and the Department of Clinical Microbiology at Rigshospitalet.

#### Body anthropometrics and composition.

Body weight and height were measured to calculate BMI, and waist and hip circumference to calculate the waist-to-hip ratio (WHR). Dual-energy X-ray absorptiometry (DXA) scans (Lunar Prodigy GE Healthcare, Madison, Wisconsin, encore software version 14, 10, 022) were used to assess total body fat percentage and lean body mass. Visceral fat volume was assessed by MRI, using all MRI slices from the centre of the intervertebral disc above lumbar vertebrae one until the intervertebral disc below lumbar vertebrae five were included. Software used: Tomovision SliceO 5.0.16.3.1.

#### Clinical parameters.

An oral glucose tolerance test (OGTT) was performed after an overnight fast, using a glucose solution consisting of 75 g of glucose (water-free) dissolved in 300 mL of water. Blood samples were drawn at 0, 15, 30, 60, 90 and 120 minutes. Eight (5.7%) samples of insulin measurements and four (2.9%) of glucose measurements were haemolysed or lost and were imputed by using a mean of the two adjacent values. The total AUC of glucose and insulin were calculated by the trapezoidal method. Hepatic liver insulin sensitivity was evaluated with mean fasting plasma insulin and mean fasting plasma glucose using homeostasis model assessment for insulin resistance (HOMA-IR) (fasting glucose x fasting insulin)/ 22.5, primarily an index of hepatic insulin resistance. Matsuda index, which is an index of whole-body insulin sensitivity, was calculated by 10,000/square root of [fasting glucose x fasting insulin] x [mean glucose x mean insulin during OGTT] [16]. Blood for glucose, insulin, HbA1c and lipid profile were analysed using standard procedures at the Department of Clinical Biochemistry, Rigshospitalet. Blood pressure measurements were performed after a 15-minute rest in the semi-supine position.

#### Physical fitness and activity.

We used graded cardiopulmonary exercise testing to establish VO_2_max [12,17] using a prediction model for starting watts and increases for each individual [18]. The Borg Rating of Perceived Exertion scale 6–20 was used to obtain self-assessed levels of exhaustion [19]. Physical activity was measured for seven consecutive days at baseline and post-intervention using an axial accelerometer-based activity monitor (AX3; Axivity, Newcastle upon Tune, UK). The R-packages devtools and jbrond/physaccel were used to find the intensity and physical activity types [20]. We had to remove one day for all due to late registration (from 5.00 am).

#### Self-assessed health.

For self-assessed health, we used the Medical Outcome Study, RAND-developed 36-Item Short Form Survey (SF-36) in the original and in the Danish version [21]. The scoring rules for the RAND 36-item health survey (Version 1.0) were used [22]. SF-36 is a well-validated and reliable instrument for assessing health-related quality of life [23]. The SF-36 has consistently demonstrated strong internal consistency, with Cronbach’s alpha coefficients typically exceeding 0.85 and good test–retest reliability, with intraclass correlation coefficients often above 0.70. Its validity and responsiveness to changes in health status have been well documented across diverse populations [24,25].

### Randomization and blinding

The patients were block-randomized 1:1 to the intervention and control groups using sealed prepacked envelopes. The randomization sequence was generated using Sealedenvelope.com with varying block sizes, as described in detail elsewhere [12]. The envelopes were prepared by an independent researcher not involved in participant enrollment or assessment. The first author (S. Jespersen) enrolled participants and opened the envelopes sequentially immediately after baseline assessments, assigning participants irreversibly to their group. Neither the patients nor the primary investigator were blinded. The third author (C.Durrer) assessed the primary outcome of liver fat-fraction blinded without prior involvement with participants or examinations, and the primary investigator assessed the secondary outcomes unblinded.

### Changes from the original protocol

The study was originally aimed to include an assessment of hepatic steatosis through percutaneous liver biopsy [12]. However, it was withdrawn as more than five patients of the first ten declined. Only one liver biopsy was performed. Additionally, the trial was stopped early before reaching the planned sample size due to slow recruitment.

### Safety and adverse events

All patients underwent a medical examination, including an electrocardiogram, conducted by the primary investigator (S. Jespersen). The exercise group was asked about adverse events (AE) after the exercise sessions. Patients’ journals were assessed for serious adverse events (SAE) during and after the intervention period and six months later. SAE was defined as death, life-threatening conditions, hospitalizations >24 hours, an extension of hospital admittance, or invalidity [26].

### Sample size considerations

The sample size calculation was based on the primary outcome [12]. We assumed a common standard deviation (SD) of −2.6 [9] and no change in the control group. An 80% chance of detecting a −2.8% between-group change in liver fat-fraction with a one-sided 0.05 significance required n = 14 in each group; we aimed to include 30 participants in total, slightly exceeding the calculated requirement, providing a small buffer against potential dropouts.

### Statistical analysis

Baseline characteristics of the participants are presented as mean (standard deviation; SD) or median (interquartile range; IQR), depending on whether the data were symmetrically distributed.

The primary analysis of the primary outcome was analysed per protocol using analysis of covariance (ANCOVA) that included follow-up outcome as the dependent variable with fixed effects for group and baseline outcome to establish the between-group differences. The adherence thresholds for the PP analysis were pre-defined in the study protocol [12]. Participants were required to complete ≥70% of the planned 36 exercise sessions and achieve ≥50% of the prescribed HRmax during the sessions to be included in the PP analysis. Secondary analysis with adjustments for age, sex, and baseline body mass index (BMI) as covariates was also performed. Mixed models were used to find the within-group differences and included both baseline and follow-up outcomes as the dependent variable and a fixed effect for group and random intercepts for participant ID. Using this model, secondary adjusted analysis (age, sex and baseline BMI) was also performed for within-group differences. For the primary outcome, an intention to treat (ITT) analysis, using a linear mixed model (both unadjusted and adjusted) was carried out. The ITT analysis included all randomized participants with available data and was conducted without imputation of missing data or use of last observation carried forward, relying on maximum likelihood estimation under the assumption that data were missing at random.

The results are presented as estimates [95% confidence intervals (CI)], and the statistical significance was set at 0.05.

The standardized mean differences and CI were calculated by dividing point estimates and confidence limits by the SD. These are presented visually in a forest plot to indicate changes favouring the exercise intervention or control.

To account for multiple comparisons, a Bonferroni-adjusted alpha was applied to the secondary outcomes. With 32 secondary outcomes, the corrected significance threshold was calculated as α = 0.05/32 = 0.0016. Both raw and Bonferroni-adjusted p-values are reported for transparency. R version 4.3.0 was used for statistical analysis, and the package lme4 was used for the mixed models [27].

### Ethics approval statement

The study was approved by The Danish Capital Region Committee on Health Research Ethics (H-21034236) and is carried out in accordance with the ethics standards of the Helsinki Declaration. All patients included in the study filled out informed written consent. The study was approved by the Danish Data Protection Agency (P-2021–569).

## Results

### Participants

**Fig 1:**
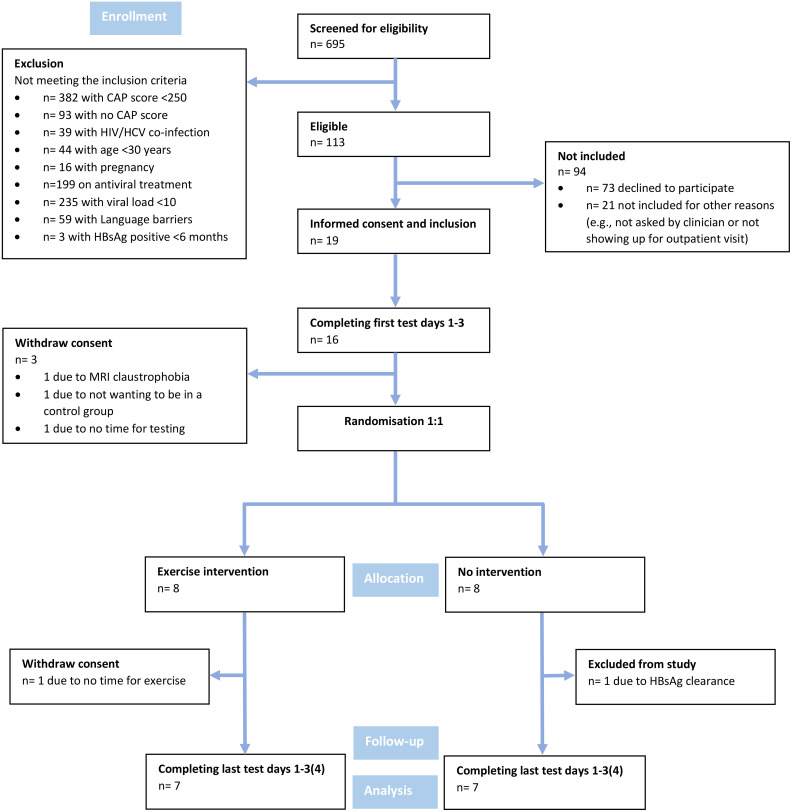
The CONSORT flow diagram.

The study inclusion period was from March 2022 until June 2023. The study included 19 out of 695 screened CHB patients, of whom 16 were randomized, including 8 in each group (Fig 1). One patient from the exercise intervention group discontinued after 2 exercise sessions, and one patient in the control group became HBsAg negative and had to be excluded. Both are included in the baseline characteristics. Baseline characteristics are presented descriptively in Table 1.

**Table 1:** Baseline characteristics of randomised participants with chronic hepatitis B and hepatic steatosis.

	All participants	Randomised to exercise	Randomised to control
Participants, n	16	8	8
Age, years	47.7 (32-68) †	49.9 (35-68) †	45.5 (32-62)†
Sex, female, n (%)	5 (45)	3 (60)	2 (33)
**Mothers’ continent of birth**			
Asia, n (%)	3 (19)	2 (25)	1 (13)
Middle East and Northern Africa (MENA) and Turkey, n (%)	7 (44)	5 (63)	2 (25)
Europe, n (%)	3 (19)	1 (13)	2 (25)
Africa, n (%)	1 (5)		1 (13)
South America, n (%)	2 (11)		2 (25)
**Liver parameters**			
Fat-fraction of Liver (MRI) %	5.8 (10.8) ‡	10.4 (10.6) ‡	5.1 (5.3) ‡
ALT, U/L	32.9 (13.0)	34.0 (13.0)	31.9 (13.7)
HBV viral load, IU/mL	385.5 (898.5) ‡	643.5 (1234.5) ‡	190 (779.3) ‡
HBV e-antigen positive, n (%)	0 (0)	0 (0)	0 (0)
Fibrosis index −4 score	0.97 (0.49)	1.07 (0.48)	0.86 (0.51)
TE score, kPa	6.05 (1.48)	5.99 (1.55)	6.11 (1.50)
CAP, dB/m	304.8 (40.8)	307 (32.8)	302.5 (49.7)
**Body anthropometrics**			
Body weight, kg	85.4 (19.1)	80.9 (19.5)	89.9 (18.8)
Body mass index, kg/m^2^	28.7 (3.9)	29.2 (4.3)	28.1 (3.7)
Hip-waist ratio	0.90 (0.12)	0.88 (0.16)	0.91 (0.08)
**Body composition**			
Lean body mass, kg	53.2 (13.6)	49.1 (13.6)	57.3 (13.2)
Body fat percentage, %	33 (8)	35 (9.7)	32 (6.1)
Visceral body fat, cm^2^	2500 (1370)	2390 (1496)	2610 (1324)
**Clinical parameters**			
Systolic blood pressure, mmHg	121.3 (11.1)	118.1 (6.3)	124.5 (14.2)
Diastolic blood pressure, mmHg	77.4 (6.1)	76.0 (5.8)	78.9 (6.5)
HbA1c, mmol/mol	36.3 (4.6)	37 (4.5)	35.5 (5.0)
Fasting glucose, mmol/L	5.2 (0.5)	5.2 (0.5)	5.2 (0.4)
2-hour glucose, mmol/L	7.0 (1.8)	7.1 (1.8)	7.0 (2.0)
Cholesterol, mmol/L	5.2 (1.5)	5.4 (1.7)	5.0 (1.2)
Triglycerides, mmol/L	1.2 (0.9) ‡	1.3 (0.7) ‡	0.8 (0.9) ‡
LDL- cholesterol, mmol/L	3.0 (1.1) ‡	3.1 (1.1) ‡	2.6 (1.1) ‡
**Physical fitness**			
VO_2_max, mL/kg/min	24.8 (7.0)	24.1 (7.6)	25.7 (6.8)

Abbreviations: ALT, alanine aminotransferase; CAP, controlled attenuation parameter; HbA1c, haemoglobin A1c; HBV, hepatitis B virus; HOMA-IR, Homeostatic Model Assessment for Insulin Resistance; MRI, magnetic resonance imaging; LDL, low-density lipoprotein; TE, transient elastography; VO_2_max, maximal oxygen consumption. Numbers are given in means (standard deviation) unless otherwise stated: †mean (range) ‡median (interquartile range).

### Primary outcome: Liver fat-fraction

**Fig 2:**
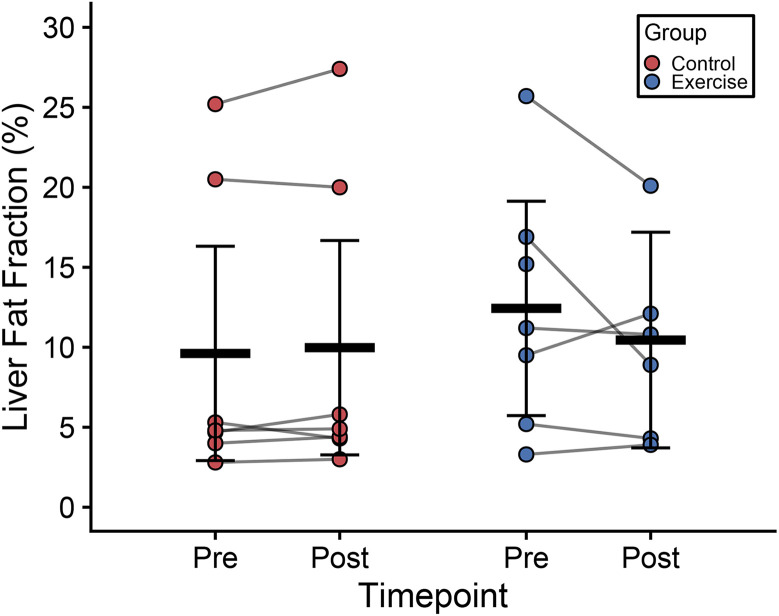
Changes in liver fat fraction from baseline to follow-up in patients with chronic hepatitis B and hepatic steatosis following the 12-week intervention. Liver Fat Fraction was assessed by magnetic resonance imaging proton density fat fraction.

The inclusion criteria of a CAP > 250 aimed at ensuring a liver fat-fraction of >5%. Six of the included patients (42%), two in the exercise group and four in the control group, had a liver fat-fraction <5% at baseline, ranging from 2.8%− 4.8% when assessed by MRI-PDFF. The patients with an MRI-PDFF liver fat-fraction >5% had CAP values ranging from 296 dB/m to 369 dB/m). One follow-up scan of an exercise group patient was missing due to technical issues. The individual data and estimated marginal means with 95% CI are shown in Fig 2. There were no significant between-group differences in the change in liver fat-fraction (Table 2). The ITT and per-protocol analyses did not differ (Supplementary Table 1 in S2 File). The adjusted ANCOVA analysis of the between-group differences in liver fat-fraction, including the variables age, sex, and BMI, showed a difference of −2.35% points [−5.26 to 0.55] with a p-value of 0.097 (Supplementary Table 1 in S2 File).

**Table 2:** Per protocol within and between-group differences of all outcomes in patients with chronic hepatitis B and hepatic steatosis following the 12-week intervention.

	Exercise groups within-group difference, n = 7	Control groups within-group difference, n = 7	Between-group differences	Between-group difference p-value	Bonferroni adjusted p-value
**Liver parameters**					
Liver fat fraction, %	−1.98 [−4.22 to 0.27]	0.36 [−1.73 to 2.44]	−2.03 [−5.49 to 1.42]	0.22	1.0
ALT, U/L	−0.71 [−6.31 to 4.88]	4.00 [−1.60 to 9.60]	−4.36 [−13.17 to 4.44]	0.30	1.0
HBV Viral load IU/mL	1904 [−917–4725]	−153 [−2974–2668]	−1.99 [−1254–1250]	0.99	1.0
Fibrosis index −4 score	−0.03 [−0.13 to 0.06]	0.02 [−0.09 to 0.12]	−0.05 [−0.22 to 0.13]	0.55	1.0
TE score, kPa	−0.47 [−1.49 to 0.55]	−1.66 [−2.74 to −0.59]	1.10 [0.53 to 1.66]	0.0048	0.15
CAP, dB/m	−31.9 [−61.4 to −2.3]	−24.3 [−55.7 to 7.1]	−6.41 [−53.96 to 41.14]	0.77	1.0
**Body anthropometrics**					
Body weight, kg	0.10 [−1.32 to 1.52]	−0.24 [−1.66 to 1.18]	0.55 [−1.87 to 2.98]	0.63	1.0
Waist-to-hip ratio	0.03 [−0.05 to 0.11]	−0.01 [−0.09 to 0.07]	0.01 [−0.08 to 0.09]	0.84	1.0
**Body composition**					
Lean body mass, kg	0.6 [−0.7 to 1.9]	−0.5 [−1.8 to 0.9]	1.2 [−1.2 to 3.6]	0.51	1.0
Total body fat, %	−0.41 [−1.91 to 1.03]	−0.18 [−1.66 to 1.29]	−0.35 [−2.84 to 2.14]	0.76	1.0
Visceral Adipose tissue, cm³	−166 [−509–177]	−29 [−347–288]	−138 [−698–420]	0.59	1.0
**Clinical parameters**					
Cholesterol, mmol/L	−0.13 [−0.55 to 0.29]	−0.33 [−0.75 to 0.09]	0.29 [−0.32 to 0.90]	0.32	1.0
Triglycerides, mmol/L	0.38 [−0.03 to 0.79]	−0.06 [−0.47 to 0.35]	0.41 [−0.22 to 1.07]	0.18	1.0
LDL-cholesterol, mmol/L	−0.33 [−0.61 to 0.05]	−0.27 [−0.55 to 0.01]	0.008 [−0.38 to 0.40]	0.96	1.0
HbA1c, mmol/mol	0.14 [−1.31 to 1.60]	0.29 [−1.17 to 1.74]	−0.26 [−2.79 to 2.27]	0.83	1.0
Fasting glucose, mmol/L	−0.29 [−0.58 to 0.01]	−0.16 [−0.45 to 0.14]	−0.12 [−0.56 to 0.32]	0.55	1.0
Glucose AUC, mmol/L x min	−4.18 [−69.35 to 60.99)	−59.68 [−124.85 to 5.49)	57.15 [−51.31 to 165.62]	0.27	1.0
Insulin AUC, pmol/L x min	−4060 [−22101–13980]	−19232 [−37273 to −1190]	15890.0 [−1946–33718]	0.08	1.0
Matsuda Index	−0.2 [−1.0 to 0.6]	0.6 [0.2 to 1.4]	−0.9 [−2.1 to 0.4]	0.18	1.0
HOMA-IR	−0.2 [−2.0 to 1.6]	−0.8 [−2.6 to 1.0]	1.0 [−0.9 to 2.8]	0.28	1.0
Blood pressure systolic, mmHg	9.71 [−1.40 to 20.83]	−2.00 [−13.11 to 9.11]	6.36 [−6.37 to 19.09]	0.29	1.0
Blood pressure diastolic, mmHg	−2.57 [−9.78 to 4.64]	−3.29 [−10.50 to 3.93]	−0.94 [−2.70 to 0.83]	0.30	1.0
**Physical fitness and activity**					
VO_2_max, mL/kg/min	4.82 [2.51 to 7.12]	−0.88 [−3.19 to 1.43]	5.6 [1.7 −9.5]	0.0015	0.048
Moderate-to-vigorous activity, min/day	−23.36[−44.07 to 2.64]	0.34 [−22.04 to 22.72]	−14.49 [−37.41 to 8.43]	0.19	1.0
Sedentary time, min/day	8.35 [−60.30 to 77.01]	−20.27 [−94.43 to 50.88]	27.46 [−93.33 to 148.26]	0.62	1.0
**Self-assessed health**					
Physical functioning	1.43 [−11.80 to 14.66]	−12.86 [−26.09 to 0.38]	10.34 [−11.66 to 32.34]	0.32	1.0
Role limitations due to physical health	−17.86 [−51.43 to 15.71]	0.00 [−33.57 to 33.57]	−11.60 [−61.90 to 38.71]	0.62	1.0
Role limitations due to emotional problems	−4.76 [−21.8 to 12.3]	−4.76 [−21.8 to 12.3]	−2.86 [−31.27 to 25.56]	0.83	1.0
Energy/fatigue	2.86 [−7.87 to 13.58]	−5.71 [−16.44 to 5.01]	10.27 [−2.96 to 23.50]	0.12	1.0
Emotional well-being	9.71 [1.66 to 17.76]	−11.43 [−19.48 to −3.38]	18.07 [6.58 to 29.56]	0.0004	0.013
Social functioning	0.0 [−20.26 to 20.26]	8.9 [−11.33 to 29.19]	−1.98 [−32.56 to 28.59]	0.89	1.0
Pain	−11.07 [−30.83 to 8.68]	−13.21 [−32.97 to 6.54]	2.12 [−30.6 to 34.85]	0.89	1.0
General health perceptions	0.00 [−13.53 to 13.53]	5.00 [−8.53 to 18.53]	−4.30 [−26.64 to 18.04]	0.68	1.0

Abbreviations: AUC, area under the curve; ALT, alanine aminotransferase; CAP, controlled attenuation parameter; HbA1c, haemoglobin A1c; HBV, hepatitis B virus; HOMA-IR, Homeostatic Model Assessment for Insulin Resistance; LDL, low-density lipoprotein; TE, transient elastography; VO_2_max, maximal oxygen consumption. Group differences are calculated by use of a multistate model with no adjustments. All data is given in mean [95% confidence intervals]. Within-group differences are calculated by use of a linear mixed model. Between-group differences are computed using the ANCOVA model. Statistical significance was determined using a Bonferroni-adjusted alpha = 0.0016 (0.05/32), all tests were two-sided, and both raw and Bonferroni-adjusted p-values (calculated as raw p-value × 32, capped at 1) are reported for transparency.

### Secondary outcomes

**Fig 3:**
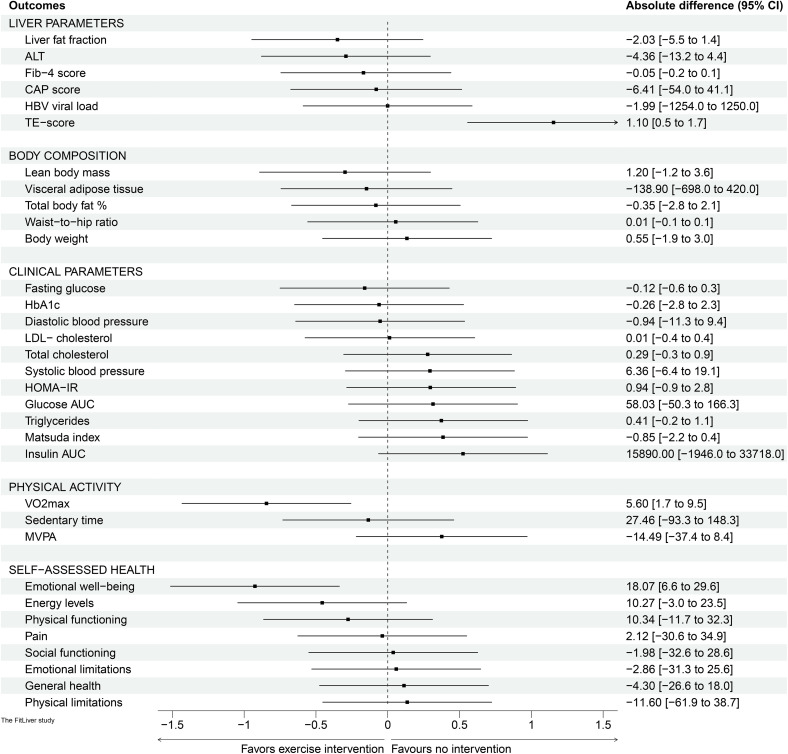
The trends of changes in all outcomes in patients with chronic hepatitis B and hepatic steatosis following the 12-week intervention. Standardised mean differences between the groups with absolute differences [95% confidence intervals] added for interpretation. Abbreviations: ALT, alanine aminotransferase; AUC, area under the curve; CAP, controlled attenuation parameter; FIB-4, Fibrosis index −4 score; HbA1c, haemoglobin A1c; HBV, hepatitis B virus; HOMA-IR, Homeostatic Model Assessment for Insulin Resistance; LDL, low-density lipoprotein; MVPA, moderate to vigorous physical activity, TE, transient elastography; VO_2_max, maximal oxygen consumption.

Secondary outcomes are presented in Table 2 and Fig 3. Between-group differences did not differ significantly regarding liver parameters (except the TE-score), body anthropometrics, body composition, clinical parameters, physical fitness and activity measures (except VO_2_max) and self-health assessment scores (except for emotional well-being). A significant decrease in the insulin AUC were seen within the control group. Changes in glucose AUC, HOMA-IR and Matsuda did not differ between groups.

### Intervention and protocol adherence

All but one patient (patient 5) fulfilled the pre-defined adherence criteria of conducting ≥ 70% of the 36 planned sessions with ≥ 50% of the assigned HRmax [12] (Supplementary Table 2 in S2 File). This participant was suspected of having an inaccurate HRmax at baseline testing since the participant exercised to exhaustion without reaching the HR goals and had an average BORG mean of 19.1 (scale 6–20) compared to the general mean of 15 in all participants. Furthermore, the participant also had high watt intensities. The patient was therefore included in the per-protocol analysis, and a supplementary analysis excluding the patient from the per-protocol analysis showed no changes in results (Supplementary Table 1 in S2 File). For attendance, we found a cancellation mean of 7 sessions/patient (range: 0–14 cancellations).

### Safety

The exercise intervention was considered well-tolerated and safe. A total of 23 AEs were reported, and the majority were assessed as mild. These included knee pain, leg cramps, exhaustion, vasovagal syncope, and vomiting. One SAE occurred in the control group, where a patient was hospitalized for one day due to abdominal pain unrelated to the study (Supplementary Tables 3 and 4 in S2 File).

## Discussion

### Overall findings

The major finding of this study was that patients with CHB and hepatic steatosis can obtain cardiovascular training adaptation. All patients had low baseline VO_2_max levels relative to age and sex-matched norms, and a significant and clinically relevant improvement in VO₂max was observed following the intervention. However, we did not observe an exercise training effect on liver fat-fraction or cardiometabolic risk factors.

These findings are consistent with some previous exercise intervention studies. A recently published study of a four-week HIIT intervention in 40 obese adolescents reported no significant changes in liver fat-fraction in the exercise group compared to the control group [28]. They used a cut-off CAP value of 241 dB/m, and only 11 persons (35%) in the exercise group fulfilled the criteria of hepatic steatosis >5% when examined by MRI-PDFF. The study was assessed to be underpowered, and in a subgroup analysis, the 11 persons with baseline MASLD had a non-significant tendency to decrease in liver fat-fraction. Taken together with our results (i.e., only 43% having hepatic steatosis with a CAP > 250 dB/m), this would suggest a need for a higher CAP threshold when assessing hepatic steatosis.

An intervention study of 12 weeks duration, by Langleite et al, involving combined resistance and endurance training exercises, resulted in reduced hepatic steatosis in dysglycemic men compared to normoglycemic men [9]. Additionally, a study by Hallsworth et al, using a HIIT intervention in patients with NAFLD showed a decrease in liver fat-fraction by 2.8% [10]. Both studies by Langleite and Hallsworth showed a decrease in body weight. In our study, the 95% CI for the change in liver fat-fraction in the exercise group was −4.22 to 0.27. This leads us to speculate that the exercise intervention might have yielded significant results had the study not been prematurely terminated and if all patients had a liver fat-fraction exceeding 5%. Other studies assessing exercise interventions on hepatic steatosis showed no decrease in hepatic steatosis: A 12-week aerobic intervention study was conducted on patients with MASLD (16 performing exercise), here liver biopsies were utilized to evaluate changes, and despite observing no significant impact on hepatic steatosis, there was a notable reduction in hepatic fibrosis stage and hepatocellular ballooning [29]. This finding of reduced fibrosis contrasts with a study involving six months of circuit exercise training in patients with steatohepatitis (13 performing exercise), which demonstrated no effect on histological steatosis and inflammation [30]. In our study, the finding of a lowered TE-score (used to assess fibrosis) within the control group at follow-up was assessed to be caused by a lack of consistent fasting at baseline [31].

### Implications for research

This study highlights several important considerations for future research. Accurate identification of hepatic steatosis at baseline is critical, and the observed discrepancy between CAP and MRI-PDFF suggests that different cut-offs or combined diagnostic approaches may be needed.

Future studies should aim for larger sample sizes to ensure adequate statistical power and consider multicenter designs, particularly in regions with higher CHB prevalence. In addition, incorporating baseline MRI-PDFF measurements and more systematic dietary monitoring would improve internal validity and interpretability.

By current evidence, we cannot say whether patients with CHB have a different response to exercise compared to patients with hepatic steatosis without CHB. It has been suggested that HBV X protein is a driver of inducing hepatic lipid accumulation [32], but the pathophysiological pathways are unclear. However, normalisation of hepatic fat content would be expected to decrease the risk of both liver cancer and all-cause mortality.

### Implications for practice

The improvement in cardiorespiratory fitness found in the study, as assessed by VO₂max, is an important finding since it is a well-established independent predictor of cardiovascular and all-cause mortality and is widely used for risk stratification and clinical decision-making across a range of conditions [33]. Additionally, higher baseline cardiorespiratory fitness has been associated with reductions in liver fat and may contribute to the resolution of hepatic steatosis independent of adiposity [34], supporting the clinical relevance of the observed improvements in VO₂max in this study.

Our study shows that the self-assessed emotional well-being was increased in the exercise group. While the between-group difference of 18 points was statistically significant, the minimally important difference (MID) for SF-36 domains in chronic diseases is highly variable, ranging from 2–4 points in idiopathic pulmonary fibrosis [35] to 13–25 points in pulmonary arterial hypertension [36], and a 3-point change is often cited as a general threshold for summary scores [37]. A study of 6,728 American people obtained improvements in emotional well-being related to the level of cardio-respiratory fitness using the General Well-Being Schedule [38], and another study reported improvements in emotional well-being, but also physical functioning, energy levels and mental health when performing exercise interventions in people with cancer [39]. As such, our results are in line with these previous findings and suggest that a similar relationship exists for patients with CHB.

### Strengths and limitations

A key strength of this study is the supervisied exercise intervention, which allows us to ensure standardized delivery and verification of training intensity as well as patient attendance. This design supports the internal validity of the intervention implementation.

However, several limitations should be acknowledged. This study is primarily limited by a small sample size and substantial heterogeneity among participants with respect to ethnicity, body weight, and liver fat fraction. Together, these factors led to wide confidence intervals, diminished statistical power, and an increased risk of type II error, thereby constraining the robustness of the conclusions. We did not succeed in achieving the target sample size, despite multiple different approaches to identify new patients to include. Due to slow recruitment, we decided to close the study before reaching the intended number of patients. This challenge is likely multifactorial. CHB has a relatively low prevalence in Denmark (approximately 0.3%) [40], which restricts the number of eligible patients available for inclusion compared to higher-prevalence regions. In addition, patient-related factors may have influenced recruitment. It has been found that many patients with CHB struggle with understanding their disease, and half of them worry about stigma when disclosing their disease [41]. This could be speculated to be the reason why they do not prioritise improving their liver status and participating in research focusing on CHB. Another important limitation of this study concerns the specificity of the screening criterion used to define hepatic steatosis. A CAP threshold >250 dB/m was applied at inclusion; however, 42% of participants had a hepatic fat fraction <5% when assessed by MRI-PDFF, the primary outcome measure. This mismatch likely reduced the study’s ability to detect intervention-related effects and may have contributed to the inconclusive findings.

The CAP cut-off was selected based on the best available evidence at the time of study design, including a meta-analysis reporting a threshold of approximately 246 dB/m [42] for the detection of hepatic steatosis, and is actually the current recommendation of using CAP to detect hepatic steatosis (>5%) in the most recent European Study of the Liver (EASL) 2024 guidelines [43]. MRI-PDFF scans were performed at both baseline and at the end of the intervention, allowing assessment of change in liver fat-fraction over time. However, baseline MRI-PDFF was not used as an eligibility criterion; instead, inclusion was based on CAP or ultrasound findings, as specified in the protocol. Consequently, the discrepancy between CAP-based screening and MRI-PDFF-defined hepatic steatosis was only identified after completion of MRI-PDFF analyses and could not be accounted for during participant selection or eligibility assessment.

In retrospect, the present results suggest that a higher CAP threshold (e.g., > 300 dB/m) may provide improved specificity when MRI-PDFF is used as the reference standard. Future studies should consider using the higher suggested CAP cut-offs or incorporating baseline MRI-PDFF measurements to better align eligibility criteria with the primary outcome. Future studies should consider applying higher CAP cut-offs or combining CAP with baseline MRI-PDFF to better align inclusion criteria with the primary outcome.

The absence of liver biopsy data further highlights the limitation of liver steatosis assessment. Although biopsies were planned, most participants declined, and only one was performed, leaving out pathological assessment of steatosis, inflammation and fibrosis.

Another limitation is the lack of systematic diet control and monitoring during the intervention. A change in diet has been shown to affect the liver fat-fraction and visceral adiposity greatly [44]. Although participants were instructed to maintain stable dietary habits throughout the study at least two patients in this study had major changes in food intake, which were not systematically or quantitively registered. These unanticipated changes, related to illness and religious lifestyle (Ramadan), may have influenced the primary and secondary outcomes and thereby reduced the ability to attribute observed effects solely to the exercise intervention. While the study was designed to examine the effects of exercise under real-world conditions without dietary restrictions, future studies would benefit from incorporating dietary monitoring or control to minimize confounding and improve interpretability.

Another consideration is that the study population primarily consisted of motivated, middle-aged patients receiving care in a Danish clinical setting, which may limit the generalizability of the findings. The results may therefore not be directly applicable to broader populations of patients with CHB, particularly in lower- and middle-income countries or in healthcare systems with different organizational structures. Nevertheless, the intervention was delivered within routine clinical care, providing pragmatic insight into its feasibility and effects in a real-world, high-income setting.

Although both intention-to-treat and per-protocol analyses were performed, the small sample size limits their robustness, and the per-protocol findings in particular should be interpreted with caution. While the primary outcome was assessed in a blinded manner, secondary outcomes were not blinded, which may have introduced measurement bias. In addition, one participant demonstrated suboptimal adherence to the prescribed training intensity yet was retained in the analyses for transparency, which may have contributed to adherence-related bias. Together, these factors further limit the interpretability and generalizability of the findings.

## Conclusion

This study is the first to investigate the effects of high-intensity interval training on hepatic steatosis in patients with CHB. Due to premature discontinuation and the resulting smaller-than-planned sample size, the study was underpowered, and the estimated effects on liver fat-fraction and secondary outcomes, such as body composition, lipid and glucose metabolism, and blood pressure should therefore be interpreted as exploratory rather than confirmatory. However, we found that the intervention was well-tolerated, with no safety concerns identified, and that it improved cardiorespiratory fitness by improving VO_2_max and self-reported emotional well-being.

## Supporting information

S1 File The CONSORT checklist. (DOCX)

S2 File Supplementary of four tables including secondary analysis, exercise compliance data, and adverse events. (DOCX)

S3 File Protocol version 1.3 25112021 without figure 2. (PDF)
