# Supplementary material for: Effect of high-intensity interval training in patients with chronic hepatitis B and hepatic steatosis: A randomised controlled trial
Source: PLoS One. 2026 Jun 23;21(6):e0351547. doi: 10.1371/journal.pone.0351547 (PMC13289930; doi:10.1371/journal.pone.0351547)
Supplement: S2 File — (DOCX) [file pone.0351547.s002.docx]

**Supplementary**

**Article:** Effect of high-intensity interval training in patients with chronic hepatitis B and hepatic steatosis: a randomised controlled trial

**Supplementary tables**

**Supplementary Table 1:** Unadjusted and adjusted per protocol and intention-to-treat analyses of liver fat fraction.

|  | **Within-group difference: exercise group** | **Within-group difference: control group** | **Between-group differences** | **P- value** |
| --- | --- | --- | --- | --- |
| PP unadjusted | -1.98 [-4.22 to 0.27] | 0.36 [-1.73 to 2.44] | -2.03 [-5.49 to 1.42] | 0.22 |
| PP adjusted | -2.03 [-4.28 to 0.22] | 0.36 [-1.73 to 2.44] | -2.35 [-5.26 to 0.55] | 0.10 |
| ITT unadjusted | -1.92 [-4.16 to 0.32] | 0.38 [-1.69 to 2.46] | -2.30 [-5.35 to 0.75] | 0.29 |
| ITT adjusted | -1.93 [-4.17 to 0.32] | 0.41 [-1.68 to 2.49] | -2.33 [-5.40 to 0.73] | 0.28 |
| **Without the participant not fulfilling PP by pre-definition** |  |  |  |  |
| PP unadjusted | -0.76 [-2.58 to 1.06] | 0.36 [-1.18 to 1.90] | -0.99 [-3.75 to 1.76] | 0.44 |
| PP adjusted | -0.79 [-2.61 to 1.03] | 0.35 [-1.19 to 1.90] | -1.5 [-4.18 to 1.10] | 0.20 |
| Log PP unadjusted | 0.99 [0.85 to 1.14] | 1.04 [0.91 to 1.17] | 1.07 [0.75 to 1.53] | 0.67 |
| Log PP adjusted | 0.98 [0.85 to 1.14] | 1.04 [0.91 to 1.17] | 0.96 [-0.25 to 0.17] | 0.67 |

In per protocol (PP) n = 7 and intention to treat (ITT) n = 8 in each group. PP uses the ANCOVA model to estimate within-group differences, and ITT uses the mixed model to estimate group differences. Adjustments are sex, age, and BMI at baseline.

**Supplementary Table 2:** Exercise compliance data

| **Variable  \  Patient** | **All** | **1** | **2** | **3** | **4** | **5** | **6** | **7** |
| --- | --- | --- | --- | --- | --- | --- | --- | --- |
| Total HIIT session attended (no.) | **33.3** | 34 | 36 | 31 | 28 | 36 | 36 | 32 |
| Total sessions not attended (no.) | **2.7** | 2 | 0 | 5 | 8 | 0 | 0 | 4 |
| Total sessions without reaching heart rate goal (no.) | **7.7** | 1 | 4 | 3 | 0 | 33 | 6 | 7 |
| Total sessions with missing heart rate data | **0.7** | 0 | 0 | 2 | 1 | 2 | 0 | 0 |
| Time in HR zone 1, (min) <70 % of HRmax | **8.2 (2.1)** | 7.4 (3.0) | 6.8 (3.9) | 7.0 (3.6) | 7.4 (4.1) | 14.5 (5.9) | 3.8 (2.9) | 10.5 (3.8) |
| Time in HR zone 2, (min) 70-74 % of HRmax | **5.5 (3.2)** | 3.1 (2.0) | 5.8 (2.4) | 3.0 (1.7) | 3.9 (2.0) | 8.8 (3.4) | 6.9 (3.0) | 6.0 (2.7) |
| Time in HR zone 3, (min) 75-79 % of HRmax | **6.2 (2.7)** | 4.7 (2.2) | 5.3 (2.5) | 5.0 (1.6) | 5.5 (2.6) | 8.0 (3.0) | 8.4 (2.3) | 5.9 (1.7) |
| Time in HR zone 4, (min) 80-84 % of HRmax | **5.9 (2.5)** | 7.1 (1.6) | 4.0 (2.0) | 5.7 (1.1) | 5.9 (2.4) | 5.0 (3.4) | 7.8 (2.6) | 5.7 (0.9) |
| Time in HR zone 5, (min) >85 % of HRmax | **11.4 (6.1)** | 15.0 (4.4) | 14.4 (5.3) | 15.3 (3.4) | 13.6 (5.8) | 2.7 (3.1) | 10.4 (4.2) | 9.9(4.4) |
| Borg scale | **15.7 (2.2)** | 13.9 (1.0) | 15.1 (2.4) | 14.7 (1.2) | 15.8 (1.8) | 19.1 (0.8) | 16.0 (1.4) | 14.8 (1.2) |
| Watts in intervals | **88.7 (58.5)** | 182.4 (6.8) | 49.7 (8.4) | 61.5 (6.4) | 31.2 (2.0) | 139.8 (7.3) | 37.0 (3.4) | 119.4 (8,4) |
| Watts in between intervals | **44.0 (29.1)** | 77.0 (8.7) | 32.3 (3.2) | 19.4 (1.5) | 17.4 (2.5) | 89.3 (9.8) | 22.6 (4.2) | 49.8 (5.4) |

All data are presented as mean (SD). Abbreviations: HIIT: High-Intensity Interval Training, HR: heart rate, HRmax: maximal heart rate, min: minutes, no: number.

**Adverse events**

**Adverse event definition**

An adverse event (AE) is defined as any untoward medical occurrence in a study participant that does not necessarily have a causal relationship with the allocated treatment (1). An AE is any unfavorable and unintended sign (including an abnormal laboratory finding), symptom, or disease temporally associated with the investigational treatment, whether the event is considered causally related to the treatment (1).

**Adverse event severity**

Definitions of the severity of each AE:

Mild    = The AE is transient and easily tolerated by the participant.

Moderate = The AE causes the participant discomfort and interrupts the participant's usual activities.

Severe        = The AE causes considerable interference with the participant's usual activities and may be incapacitating or life-threatening.

**Serious adverse events definition**

•                Results in death

•                Is life-threatening

•                Requires inpatient hospitalization or prolongation of existing hospitalization

•                Results in persistent or significant disability/incapacity

•                Is a congenital anomaly/birth defect

•                Is a medically important event

**Supplementary Table 3:** Adverse events in the exercise group

|  | **Adverse event**  (Number of participants with an event) | | | **SAE** | **Assessed related to the study** |
| --- | --- | --- | --- | --- | --- |
| **Event** | **Mild** | **Moderate** | **Severe** |  |  |
| Knee pain | 2 |  |  |  | Yes |
| Back pain | 1 |  |  |  | No |
| Hip pain | 2 |  |  |  | No |
| Leg cramp | 1 |  |  |  | Yes |
| Dyspnea | 2 |  |  |  | No |
| Pneumonia |  | 2 |  |  | No |
| Exhaustion | 2 |  |  |  | Yes |
| Tiredness | 3 |  |  |  | No |
| Gastrointestinal infection |  | 1 |  |  | No |
| Vasovagal syncope ^ | 1 |  |  |  | Yes |
| Vomiting ^ | 1 |  |  |  | Yes |
| Insomnia |  | 1 |  |  | No |
| **TOTAL** | **15** | **4** | **0** | **0** |  |

Abbreviations: SAE = serious adverse event. ^ The event occurred just after finishing the 4^th^ interval in an exercise session

**Supplementary Table 4:** Adverse events in the control group

|  | **Adverse event**  (Number of participants with an event) | | | **SAE** | **Assessed related to the study** |
| --- | --- | --- | --- | --- | --- |
| **Event** | **Mild** | **Moderate** | **Severe** |  |  |
| Gastrointestinal infection |  | 1 |  |  | No |
| Vasovagal syncope | 1 |  |  |  | Yes |
| Insomnia |  | 1 |  |  | No |
| Abdominal pain * |  |  |  | 1 | No |
| **TOTAL** | **1** | **2** |  | **1** |  |

Abbreviations: SAE = serious adverse event. * Refers to hospital admittance lasting 1 day, which happened 8 days after a liver biopsy.

**List of references**

1. International Council for Harmonisation of Technical Requirements for Registration of Pharmaceuticals for Human Use. ICH Topic E 2 A Clinical Safety Data Management: Definitions and Standards for Expedited Reporting -Scientific guideline [Internet]. 1995 Jun. Available from: https://www.ema.europa.eu/en/ich-e2a-clinical-safety-data-management-definitions-and-standards-expedited-reporting-scientific-guideline
