# Supplementary material for: Effect of high-intensity interval training in patients with chronic hepatitis B and hepatic steatosis: A randomised controlled trial
Source: PLoS One. 2026 Jun 23;21(6):e0351547. doi: 10.1371/journal.pone.0351547 (PMC13289930; doi:10.1371/journal.pone.0351547)
Supplement: S3 File — (PDF) [file pone.0351547.s003.pdf]

Effect of aerobic exercise training on fat-fraction of the liver in patients with chronic hepatitis B and hepatic steatosis: a randomised controlled intervention trial. The Fit Liver study

(Effekt af konditionstræning på fedtprocenten i leveren hos patienter med kronisk hepatitis B og fedtlever)

THE CAPITAL REGION'S COMMITTEE ON HEALTH RESEARCH ETHICS

Protocol version 1.30

Principal Investigator

Sofie Dikeledi Kold Jespersen, MD, PhD student,
Center for Physical Activity Research, Copenhagen University Hospital, Rigshospitalet
Ole Maaløes Vej 24, 2200 Copenhagen N
Phone: +45 2666 5112
Mail: sofie.dikeledi.kold.jespersen@regionh.dk

Funding:

The Centre for Physical Activity Research (CFAS) is supported by TrygFonden (grants ID 101390 and ID 20045). A grant from the Beckett Foundation and Holms Mindelegat further supported the study

Places of Execution

Department of Infectious Diseases, Copenhagen University Hospital -Hvidovre, Kettegaard Alle 30, 2650 Hvidovre

Centre for Physical Research Activity (CFAS), Copenhagen University Hospital -Rigshospitalet, Blegdamsvej 9, 2100 Kbh Ø

Co-Investigators

Nina Weis, Professor, PhD, senior physician, Department of Infectious Diseases, Copenhagen University Hospital -Hvidovre

Rikke Krogh-Madsen, Clinical Associate Professor, PhD, senior physician, Department of Infectious Diseases, Copenhagen University Hospital -Hvidovre and CFAS, Rigshospitalet, University of Copenhagen, Denmark

Peter Plomgaard, Clinical Associate Professor, PhD, senior physician, Department of Clinical Biochemistry, Copenhagen University Hospital, Rigshospitalet

Bente Klarlund Pedersen, Professor, Dr. Med. Sci., senior physician, Center for Physical Activity Research, Copenhagen University Hospital, Rigshospitalet

Jan Gerstoft, Professor, Dr. Med. Sci., senior physician, Department of Infectious Diseases, Copenhagen University Hospital, Rigshospitalet

Anne-Mette Lebech, Clinical Associate Professor, Dr. Med. Sci., PhD, senior physician, Department of Infectious Diseases, Copenhagen University Hospital, Rigshospitalet

Sten Madsbad, Professor, Chief Physician, MD, Dr.Med.Sci., Department of Endocrinology, Copenhagen University Hospital - Hvidovre

1: ORIGINAL TITLE

Effect of physical exercise on fat-fraction of the liver in patients with chronic hepatitis B and hepatic steatosis: a randomised controlled intervention trial.

2: AIM

Overall study aim: To investigate whether regular aerobic exercise training will decrease the fat-fraction of the liver in patients with chronic hepatitis B (CHB) and hepatic steatosis.

Secondary aim: To investigate the effects of aerobic exercise training on hepatokine secretion in patients with CHB and hepatic steatosis. Also, to investigate if regular physical exercise will reduce obesity and improve lipid- and glucose metabolism, liver status -such as elevated ALT and INR, markers of inflammation, body composition, and blood pressure.

Hypothesis: Regular aerobic exercise training decreases the fat-fraction of the liver and conditions of the metabolic syndrome in patients with CHB. Exercise-induced hepatokine release, glucose, and lipid metabolism are compromised in patients with CHB and hepatic steatosis and benefits from aerobic exercise training.

Primary endpoint:

Effect of aerobic exercise on:

- Fat-fraction of the liver assessed with magnetic resonance spectroscopy.

Secondary endpoints:

Effects of aerobic exercise on:

- Hepatokine/cytokine secretion
- Markers of lipid and glucose metabolism
- Liver status
- Body composition
- Blood pressure

Background:

Hepatitis B virus (HBV) causes CHB, which is defined as hepatitis B surface antigen (HBsAg) in blood > 6 months. The global prevalence of CHB is more than 250 million individuals (1), and CHB is the most common type of hepatitis in the world. Untreated, CHB can lead to the development of liver cirrhosis and -cancer, known as hepatocellular carcinoma (HCC). There is at present no curable therapy, but medical treatment can reduce the amount of virus in the blood, leading to reduced CHB-induced morbidity and mortality.

Comorbidity in CHB patients

CHB patients have a higher body mass index (BMI), consume more alcohol, and have poorer physical fitness compared to uninfected (2), and approximately 33 % are overweight and 50 % physically inactive (3). In general, obesity is associated with metabolic syndrome (i.e. central obesity, hypertension, glucose abnormality, and dyslipidemia) and thereby metabolic diseases such as type 2 diabetes (T2DM) and cardiovascular disease (CVD) (4). Metabolic syndrome is also correlated with non-alcoholic fatty liver disease (NAFLD). NAFLD is projected to have a prevalence of >30 % in Asia and the USA (5) (6). NAFLD is defined by the presence of steatosis >5 % in the hepatocytes (7) but excludes the diagnosis of hepatitis B. Hence this study will refer to the term hepatic steatosis when describing fatty liver disease in patients with CHB. In persons with obesity and CHB infection, the development of hepatic steatosis imposes a double burden on the liver. CHB infection and hepatic steatosis have been shown to increase the risk of cirrhosis and HCC (8) (9).

Systemic inflammation in patients with CHB and comorbidity

Obesity is associated with chronic systemic low-grade inflammation, which is the key point in the initiation and progression of obesity-related NAFLD (10) (11) (12). Chronic systemic low-grade inflammation is characterised by a two- to fourfold elevation in circulating levels of inflammatory cytokines and acute-phase reactants (13). One of the main mechanisms is thought to be increased inflammation of the adipose tissue (14). Circulating levels of tumour necrosis factor (TNF)-alpha and interleukin (IL)-6 are increased in chronic HBV infection and are positively associated with disease progression (hepatitis B progression to cirrhosis or HCC) (15) (16). Whether chronic inflammation is due to HBV infection, comorbidities like obesity and T2DM, or both are unclear (17).

Hepatokines in patients with CHB

Another unclear mechanism is the signalling pathways of hepatokines. Hepatokines are proteins secreted by hepatocytes, and several hepatokines have been associated with metabolic dysfunctions (18) (19). Hepatokines are suggested to change during HBV infection. Increased plasma levels of the hepatokine Fibroblast Growth Factor 21 (FGF21) are associated with hepatic steatosis (20), but low levels of FGF21 are associated with advanced fibrosis/cirrhosis in patients with CHB. FGF-21 is regulated by glucagon and is increased after exercise (21). Another hepatokine, follistatin, plays a critical role in hepatocyte regeneration during the repair of liver tissue (22) and is also a part of the process of muscle hypertrophy (23). Follistatin has an inverse correlation with HBV DNA levels (24). The hepatokine ANGPTL4 is secreted from the liver during exercise (25) and is involved in regulating plasma triglyceride levels (26). No studies have yet explored ANGPTL4´s relation to CHB. Studies investigating the secretion of the hepatokines are warranted in order to understand how the pathways are regulated and which target areas future medical treatment should focus on.

Effects of exercise

Exercise interventions of 6 weeks or more show beneficial effects on NAFLD through improvements in fat-fraction of the liver (27) (28), insulin resistance, liver fatty acid metabolism, and activation of inflammatory cascades after the physical exercise intervention (29). The anti-inflammatory properties of exercise are multifactorial. At least due to both a reduction in visceral adipose tissue and more direct induction of anti-inflammatory, immune cells.

IL-6 is a cytokine implicated in the regulation of energy metabolism (30) (31). IL-6 can be persistently elevated in individuals with obesity (32) and acutely following infection (33) and exercise (34) (35).

In exercise settings, IL-6 is a myokine produced by contracting muscle fibres and released into the bloodstream (35) (36). It has been suggested that IL-6 could suppress HBV replication and inhibit HBV entry (36) (37). The possible beneficial effect of the exercise-induced increase in circulating IL-6 in chronic HBV infection is unknown.

It is essential to clarify the role of physical exercise in preventing developing comorbidity and treating lifestyle-induced conditions like obesity and fatty liver disease. Furthermore, it is necessary to understand the metabolic mechanism of activating inflammatory cascades and signalling through cytokines and hepatokines to improve targeting treatment for the double burden chronically diseased persons. To our knowledge, no randomised exercise intervention study has ever been made in CHB patients. We wish to

conduct a randomised controlled intervention trial to investigate the effects of physical exercise on CHB patients with hepatic steatosis to investigate the changes in fat-fraction of the liver and how physical exercise alternates conditions related to the metabolic syndrome and cytokine and hepatokine secretion.

Literature references:

1. World Health Organization. Global Hepatitis Report 2017. 2017.
2. Chen Y-J, Chen K-W, Shih Y-L, Su F-Y, Lin Y-P, Meng F-C, m.fl. Chronic hepatitis B, nonalcoholic steatohepatitis and physical fitness of military males: CHIEF study. World J Gastroenterol. 2017;23(25):4587.
3. Yi Y-H, Kim Y-J, Lee S-Y, Cho B-M, Cho Y-H, Lee J-G. Health behaviors of Korean adults with hepatitis B: Findings of the 2016 Korean National Health and Nutrition Examination Survey. World J Gastroenterol. 28. juli 2018;24(28):3163–70.
4. Cefalu WT, Bray GA, Home PD, Garvey WT, Klein S, Pi-Sunyer FX, m.fl. Advances in the science, treatment, and prevention of the disease of obesity: Ref lections from a diabetes care editors' expert forum. Diabetes Care. 1. august 2015;38(8):1567–82.
5. Estes C, Razavi H, Loomba R, Younossi Z, Sanyal AJ. Modeling the epidemic of nonalcoholic fatty liver disease demonstrates an exponential increase in burden of disease: Estes et al. Hepatology. januar 2018;67(1):123–33.
6. Li J, Zou B, Yeo YH, Feng Y, Xie X, Lee DH, m.fl. Prevalence, incidence, and outcome of non-alcoholic fatty liver disease in Asia, 1999–2019: a systematic review and meta-analysis. Lancet Gastroenterol Hepatol. maj 2019;4(5):389–98.
7. European Association for the Study of the Liver (EASL), European Association for the Study of Diabetes (EASD), European Association for the Study of Obesity (EASO). EASL-EASD-EASO Clinical Practice Guidelines for the management of non-alcoholic fatty liver disease. Diabetologia. juni 2016;59(6):1121–40.
8. Chan AWH, Wong GLH, Chan H-Y, Tong JHM, Yu Y-H, Choi PCL, m.fl. Concurrent fatty liver increases risk of hepatocellular carcinoma among patients with chronic hepatitis B. J Gastroenterol Hepatol. marts 2017;32(3):667–76.
9. Portillo-Sanchez P, Bril F, Maximos M, Lomonaco R, Biernacki D, Orsak B, m.fl. High Prevalence of Nonalcoholic Fatty Liver Disease in Patients With Type 2 Diabetes Mellitus and Normal Plasma Aminotransferase Levels. J Clin Endocrinol Metab. juni 2015;100(6):2231–8.
10. Crunkhorn S. Metabolic disorders: Breaking the links between inflammation and diabetes. Nat Rev Drug Discov. april 2013;12(4):261.
11. Navab M, Gharavi N, Watson AD. Inflammation and metabolic disorders. Curr Opin Clin Nutr Metab Care. juli 2008;11(4):459–64.
12. Hotamisligil GS. Inflammation and metabolic disorders. Nature. 14. december 2006;444(7121):860–7.

13. Asghar A, Sheikh N. Role of immune cells in obesity induced low grade inflammation and insulin resistance. Bd. 315, Cellular Immunology. Academic Press Inc.; 2017. s. 18–26.

14. Grant RW, Dixit VD. Adipose tissue as an immunological organ. Obesity. 1. marts 2015;23(3):512–8.

15. Tang S, Liu Z, Zhang Y, He Y, Pan D, Liu Y, m.fl. Rather than Rs1800796 polymorphism, expression of interleukin-6 is associated with disease progression of chronic HBV infection in a Chinese Han population. Dis Markers. 2013;35(6):799–805.

16. Kiki I, Yilmaz O, Erdem F, Gundogdu M, Demircan B, Bilici M. Tumour necrosis factor-alpha levels in hepatitis B virus-related chronic active hepatitis and liver cirrhosis and its relationship to Knodell and Child-Pugh scores. Int J Clin Pract. september 2006;60(9):1075–9.

17. Zhang J, Lin S, Jiang D, Li M, Chen Y, Li J, m.fl. Chronic hepatitis B and non-alcoholic fatty liver disease: Conspirators or competitors? Liver Int Off J Int Assoc Study Liver. marts 2020;40(3):496–508.

18. Meex RCR, Watt MJ. Hepatokines: linking nonalcoholic fatty liver disease and insulin resistance. Nat Rev Endocrinol. september 2017;13(9):509–20.

19. Stefan N, Häring H-U. The role of hepatokines in metabolism. Nat Rev Endocrinol. marts 2013;9(3):144–52.

20. Mak L, Lee C, Cheung K, Wong DK, Liu F, Hui RW, m.fl. Association of adipokines with hepatic steatosis and fibrosis in chronic hepatitis B patients on long-term nucleoside analogue. Liver Int. juli 2019;39(7):1217–25.

21. Kim KH, Kim SH, Min Y-K, Yang H-M, Lee J-B, Lee M-S. Acute exercise induces FGF21 expression in mice and in healthy humans. PloS One. 2013;8(5):e63517.

22. Kogure K, Omata W, Kanzaki M, Zhang YQ, Yasuda H, Mine T, m.fl. A single intraportal administration of follistatin accelerates liver regeneration in partially hepatectomised rats. Gastroenterology. april 1995;108(4):1136–42.

23. Mendell JR, Sahenk Z, Malik V, Gomez AM, Flanigan KM, Lowes LP, m.fl. A phase 1/2a follistatin gene therapy trial for becker muscular dystrophy. Mol Ther J Am Soc Gene Ther. januar 2015;23(1):192–201.

24. Patella S, Phillips DJ, de Kretser DM, Evans LW, Groome NP, Sievert W. Characterization of serum activin-A and follistatin and their relation to virological and histological determinants in chronic viral hepatitis. J Hepatol. april 2001;34(4):576–83.

25. Ingerslev B, Hansen JS, Hoffmann C, Clemmesen JO, Secher NH, Scheler M, m.fl. Angiopoietin-like protein 4 is an exercise-induced hepatokine in humans, regulated by glucagon and cAMP. Mol Metab. oktober 2017;6(10):1286–95.

26. Yoshida K, Shimizugawa T, Ono M, Furukawa H. Angiopoietin-like protein 4 is a potent hyperlipidemia-inducing factor in mice and inhibitor of lipoprotein lipase. J Lipid Res. november 2002;43(11):1770–2.

27. Langleite TM, Jensen J, Norheim F, Gulseth HL, Tangen DS, Kolnes KJ, m.fl. Insulin sensitivity, body composition and adipose depots following 12 w combined endurance and strength training in dysglycemic and normoglycemic sedentary men. Arch Physiol Biochem. oktober 2016;122(4):167–79.

28. Hallsworth K, Thoma C, Hollingsworth KG, Cassidy S, Anstee QM, Day CP, m.fl. Modified high-intensity interval training reduces liver fat and improves cardiac function in non-alcoholic fatty liver disease: a randomised controlled trial. Clin Sci Lond Engl 1979. december 2015;129(12):1097–105.

29. Whitsett M. Physical activity as a treatment of non-alcoholic fatty liver disease: A systematic review. World J Hepatol. 2015;7(16):2041.

30. Wallenius V, Wallenius K, Ahrén B, Rudling M, Carlsten H, Dickson SL, m.fl. Interleukin-6-deficient mice develop mature-onset obesity. Nat Med. januar 2002;8(1):75–9.

31. Theurich S, Tsaousidou E, Hanssen R, Lempradl AM, Mauer J, Timper K, m.fl. IL-6/Stat3-Dependent Induction of a Distinct, Obesity-Associated NK Cell Subpopulation Deteriorates Energy and Glucose Homeostasis. Cell Metab. 5. juli 2017;26(1):171-184.e6.

32. Cottam DR, Mattar SG, Barinas-Mitchell E, Eid G, Kuller L, Kelley DE, m.fl. The chronic inflammatory hypothesis for the morbidity associated with morbid obesity: Implications and effect of weight loss. Bd. 14, Obesity Surgery. 2004. s. 589–600.

33. Ma L, Zhang H, Yin Y ling, Guo W zhi, Ma Y qun, Wang Y bo, m.fl. Role of interleukin-6 to differentiate sepsis from non-infectious systemic inflammatory response syndrome. Cytokine. 1. december 2016;88:126–35.

34. Ostrowski K, Rohde T, Zacho M, Asp S, Pedersen BK. Evidence that interleukin-6 is produced in human skeletal muscle during prolonged running. J Physiol. 1. maj 1998;508 (Pt 3):949–53.

35. Steensberg A, van Hall G, Osada T, Sacchetti M, Saltin B, Klarlund Pedersen B. Production of interleukin-6 in contracting human skeletal muscles can account for the exercise-induced increase in plasma interleukin-6. J Physiol. 15. november 2000;529 Pt 1:237–42.

36. Xia C, Liu Y, Chen Z, Zheng M. Involvement of Interleukin 6 in Hepatitis B Viral Infection. Cell Physiol Biochem Int J Exp Cell Physiol Biochem Pharmacol. 2015;37(2):677–86.

37. Palumbo GA, Scisciani C, Pediconi N, Lupacchini L, Alfalate D, Guerrieri F, m.fl. IL6 Inhibits HBV Transcription by Targeting the Epigenetic Control of the Nuclear cccDNA Minichromosome. PloS One. 2015;10(11):e0142599.

38. Christensen RH, Wedell-Neergaard A-S, Lehrskov LL, Legård GE, Dorph EB, Nymand S, m.fl. The role of exercise combined with tocilizumab in visceral and epicardial adipose tissue and gastric emptying rate in abdominally obese participants: protocol for a randomised controlled trial. Trials. 2. maj 2018;19(1):266.

39. Moghiseh M, Dept., of Occupational Health, School of Public Health, Isfahan University of Medical Sciences, Isfahan, Iran., Habibi E, Dept., of Occupational Health, School of Health, Isfahan University of Medical Science, Isfahan, Iran., Aramesh N, Inorganic Chemistry, Yasuj University, Yasuj, Iran., m.fl. The association between VO2max and heart rate of casting industry workers. J Occup Health Epidemiol. 1. januar 2013;2(1):20–6.

40. Helgerud J, Høydal K, Wang E, Karlsen T, Berg P, Bjerkaas M, m.fl. Aerobic High-Intensity Intervals ½½

41. Halson SL. Monitoring training load to understand fatigue in athletes. Sports Med Auckl NZ. november 2014;44 Suppl 2:S139-147.

42. Garber CE, Blissmer B, Deschenes MR, Franklin BA, Lamonte MJ, Lee I-M, m.fl. American College of Sports Medicine position stand. Quantity and quality of exercise for developing and maintaining cardiorespiratory, musculoskeletal, and neuromotor fitness in apparently healthy adults: guidance for prescribing exercise. Med Sci Sports Exerc. juli 2011;43(7):1334–59.

43. Halson SL. Monitoring training load to understand fatigue in athletes. Sports Med Auckl NZ. november 2014;44 Suppl 2:S139-147.

44. Pedersen BK. Anti-inflammatory effects of exercise: role in diabetes and cardiovascular disease. Eur J Clin Invest. august 2017;47(8):600–11.

45. Hansen JS, Clemmesen JO, Secher NH, Hoene M, Drescher A, Weigert C, m.fl. Glucagon-to-insulin ratio is pivotal for splanchnic regulation of FGF-21 in humans. Mol Metab. august 2015;4(8):551–60.

46. Kirkegaard-Klitbo DM, Danielsen KV, Hanson LG, Gluud LL, Siebner HR, Bendtsen F, m.fl. [Magnetic resonance imaging for the diagnosis of non-alcoholic fatty liver disease]. Ugeskr Laeger. 25. februar 2019;181(9).

47. Kleiner DE, Brunt EM, Van Natta M, Behling C, Contos MJ, Cummings OW, m.fl. Design and validation of a histological scoring system for nonalcoholic fatty liver disease. Hepatol Baltim Md. juni 2005;41(6):1313–21.

48. Rockey DC, Caldwell SH, Goodman ZD, Nelson RC, Smith AD, American Association for the Study of Liver Diseases. Liver biopsy. Hepatol Baltim Md. marts 2009;49(3):1017–44.

3: METHOD

Design and method of analysis: This study is designed as a randomised, controlled, unblinded, clinical intervention trial consisting of 12 weeks of aerobic exercise training. Thirty CHB patients with hepatic steatosis are randomised to either aerobic exercise training (intervention group, n=15) or no intervention (control group, n=15). Please see Figure 1.

Figure 1: Flowchart of participants

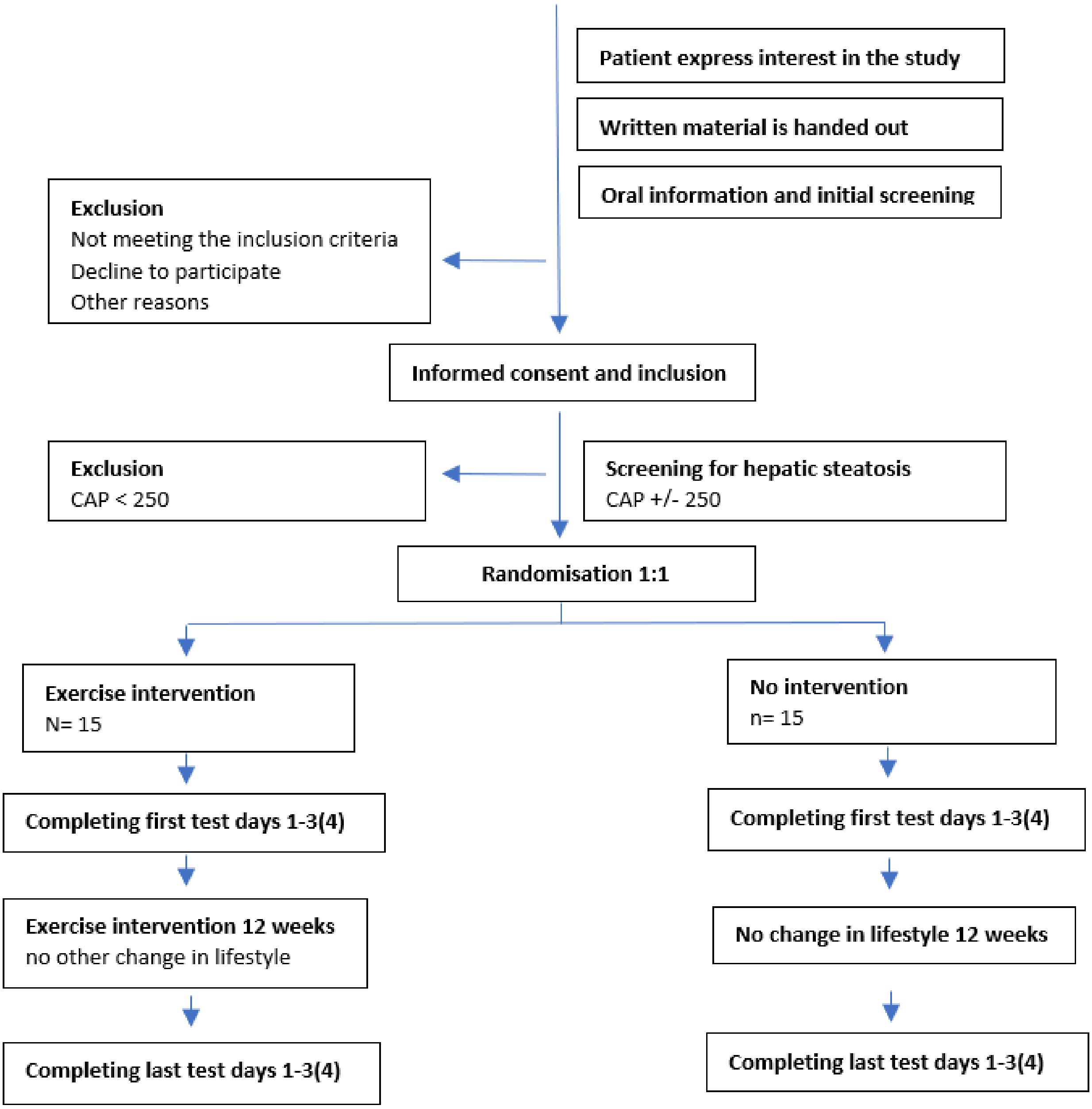

Practical implementation:

Preliminary study: clinical examination by a medical doctor or medical student, blood sampling and completion of the inclusion questionnaire (see appendix 1). Before and after the intervention, participants will undergo 3 (potentially 4, if liver biopsy is accepted) identical test days as shown in table 1:

Table 1:

Day:	Day 1	Day 2	Day 3	Day 4 (optional)
Location:	Rigshospitalet	Rigshospitalet	Rigshospitalet	Hvidovre
Hour:				
08:00 -09:00	Clinical examination and DXA scan	Hormone infusion mimicking an acute exercise bout		Clinical examination
09:00-10:00	Blood sampling and OGTT		MRS liver and MRI visceral fat	Liver biopsy
10:00-11:00				Rest and observation 5 hours
11:00-12:00				
12:00-13:00	$VO2_{max}$			
13:00-14:00				
14:00-15:00				
15:00-16:00				Clinical examination and discharge

Table 1 abbreviations: DXA: Dual-energy x-ray absorptiometry. OGTT: oral glucose tolerance test. $VO2_{max}$: maximal oxygen consumption. MRS: magnetic resonance spectroscopy. MRI: magnetic resonance imaging.

Follow-up: will be done at 12 months after the intervention has finished. It will include Day 1 and Day 3 of the test days shown in the scheme above. Six months after the intervention a brief follow -up with continuous glucose and activity monitoring will be done.

Day 1 and day 2 will be performed at the Centre for Physical Activity Research (CFAS), Copenhagen University Hospital – Rigshospitalet. Day 3 will be performed at the Department of Radiology at Copenhagen University Hospital - Rigshospitalet. Day 4, the liver biopsy (not mandatory) will be performed at the Department of Infectious Diseases, Copenhagen University Hospital – Hvidovre.

Clinical examination: This will be conducted by a medical doctor or medical student (with a medical doctor as back-up) and include heart and lung stethoscopy, EKG, blood pressure, weight, height, waist circumference and BMI measurement. It will be done before and after the intervention and at follow-up 12 months after the intervention.

Maximal oxygen consumption rate ($VO2_{max}$) Is determined at baseline, at 4 and 8 weeks, after the intervention and at follow-up 12 months after the intervention for each participant.

Aerobic exercise training: The exercise program includes three weekly supervised aerobic exercise sessions of 45 minutes/session over 12 weeks. The program consists of high-intensity endurance training on ergometer bicycles. The intensity will progress throughout the 12 weeks of training. The training consists of

8 minutes of warm-up at 40-60% maximum heart rate (HR_{max}), followed by 27 minutes of high-intensity interval training, and finally a 10 min cool-down of 50% HR_{max}. Previous studies in CFAS have had significant results following this training program (38). The maximal oxygen consumption rate ($VO2_{max}$) will be performed pre -and post-exercise/no exercise intervention and will determine relative workload accordingly.
All sessions will be supervised and administered by CFAS personnel (students of sports science or physiotherapy) or local exercise instructors. To ensure proper intensity, all patients will be wearing a heart rate monitor. Since there is a good correlation between $VO2_{max}$ and HR_{max} (39), this is a practical method to monitor the internal training load. The maximal heart rate (HR_{max}) will be measured as the peak heart rate during the final minute of the $VO2_{max}$ test (40). Heart rate monitoring is one of the most used methods to monitor internal training load (41). To evaluate the precise amount of exercise completed for every patient, each training session will be documented (attendance, total time of HR_{max} > 85%, total time of exercise, average wattage and average heart rate). The exercise programme follows the current ACSM guidelines for exercise (42) and is chosen because the exercise of similar intensity and duration is known to result in IL-6 release (43) (42) and lead to changes in VAT (42) (44). TechnoGym equipment (Pedan A/S, Copenhagen, Denmark) and software programs which allow monitoring and registration of all exercise sessions are used. To control factors that may influence the intervention's effect, the participants are instructed not to change their lifestyle habits throughout the intervention. The exercise sessions will be carried out at the Centre for Physical Activity Research (CFAS), Copenhagen University Hospital – Rigshospitalet or at the Department of Physiotherapy at Copenhagen University Hospital -Hvidovre.

Physical activity: This is measured three consecutive days before the intervention, at eight weeks, right after the intervention and at follow-up 6 - and 12 months after the intervention. For physical activity monitoring, an axial accelerometer-based activity monitor (AX3; Axivity, Newcastle upon Tune, UK) will be used.

Diet: A self-report diet form is collected before and just before the end of the intervention.

Plasma samples: Following an overnight fast (10 hours), blood samples are collected and processed by a trained laboratory technician and analysed according to standard procedures. Plasma is stored at − 80 °C before analysis. Blood samples will be analysed for: fasting glucose, fasting insulin, lipid status, Free fatty acids, triglyceride, HbA1c, haemoglobin, leucocytes and differential count, thrombocytes, CRP, ALT, AST, basic phosphatase, GGT, LDH, bilirubin, INR, FiB4, creatinine, eGFR, carbamide, Na+, K+, creatine kinase,

Transferrin saturation, reticulocytes, ferritins, IgG, IgA, GMA, anti-TG2, ferroxidase+ cobber, alpha-1-antitrypsine, anti-TPO, TSH, T3, T4, SUPAR, Amylase, Albumin, Erythrocyte vol, fr.b. , MCV, MCHC, Hepatitis A virus Ab, Hepatitis D virus Ab, Hepatitis C virus Ab, Hep B c-Ab, Hep B c-AB (IgM), Hep B virus (DNA), Hep B virus e-AB, Hep B virus e (Ag), Hep B virus DNA genotype, Hep B virus s Ab, Hep B virus a (Ag), HIV 1, HIV 2 and whole blood, purified for Peripheral Blood Monocyte Cells (PBMC). Blood samples are sampled at baseline, at 4 weeks, 8 weeks, after the intervention and at follow-up 12 months after the intervention.

Continuous glucose monitoring (CGM): Is performed using enzyme-coated electrodes (iPro MMT-7745WW; Medtronic, Northridge, CA, USA) placed subcutaneously in the upper arm. For calibration of the CGM system, the participant performs finger-prick blood glucose measurements four times daily. CGM will be done before, at week eight, after the intervention and at follow-up 6 and 12 months after the intervention.

Hormone infusion: To mimic an acute exercise bout, hormone infusion, increasing the glucagon/insulin ratio, is performed to measure the effect on circulating hepatokines. An antecubital venous catheter is placed for infusion of somatostatin (Octreotide, Hospira Nordic AB, Stockholm, Sweden) at 100 ng/kg/min., 2 hours of infusion. After 10 minutes of infusion, glucagon infusion is initiated (6 ng/kg/min, 1 h of infusion) (45). For blood sampling, an antecubital venous catheter is placed in the contralateral arm. Blood samples will be taken at baseline, 30, 60, 120, 180, 240 and 300 minutes after initiation of infusion. Blood glucose will be measured every 30th minute during the first 180 minutes. Plasma samples are collected in EDTA-containing tubes and immediately centrifuged for 15 min at 3500 g. The plasma will be stored at −80°C until further analysis of cytokines, metabolomics and glucoregulatory hormones. The hormone infusion will be done at baseline and after the intervention.

Oral glucose tolerance test (OGTT): This will be performed after an overnight fast. One intravenous catheter is placed in the anterior cubital region. After the venous baseline blood samples are drawn, the participant will drink a glucose solution consisting of 75 g of glucose (water-free) dissolved in 300 mL of water and rest for 3 hours while blood samples will be drawn at 20, 40, 60, 120 and 180 minutes. Plasma samples are collected in EDTA-containing tubes and immediately centrifuged for 15 min at 3500g, and the plasma will be stored at −80°C until further analysis. The test will be done before and after the intervention and at follow-up 12 months after the intervention.

Body composition: Dual-energy x-ray absorptiometry (DEXA) is used to assess total body fat mass and lean body mass before and after the intervention and at follow-up 12 months after the intervention.

Transient Elastography (TE): Uses ultrasound at the top of a vibrating inducer. The vibrator induces shear waves intercostal in the length of the probe, and the ultrasound echo measures the speed of the shear-waves. The TE scans will be performed at the Department of Infectious Diseases, Copenhagen University Hospital – Hvidovre before and after the intervention and at follow-up 12 months after the intervention. For a most correct scan, patients must fast 5 hours prior to the scan.

Magnetic Resonance Spectroscopy (MRS) and Magnetic Resonance Imaging (MRI): MRS of the liver is a reliable method of diagnosing and quantifying hepatic steatosis and can detect steatosis even <5 % of the liver tissue (46). MRI scans of the visceral fat will also be performed. Scans will be performed at the MRI department at Copenhagen University Hospital - Rigshospitalet. Before the examination, an MRI control scheme is filled out. If any contraindications for the scan is found, the patient is excluded from the study. If the patient has any known kidney disease, a blood test will be taken for routine analysation of kidney function before the scan. The scan will be done before and after the intervention and at follow-up 12 months after the intervention.

Liver biopsy: This is performed under hospitalisation in the Department of Infectious Diseases, Copenhagen University Hospital -Hvidovre, and is considered the gold standard for diagnosing liver inflammation and fatty liver disease. It will be used to make a detailed description of the liver tissue. The biopsy will be taken with ultrasound guidance after current instructions for planned liver biopsy (appendix 2). The procedure will take place at Function and Diagnostic Imaging Department at Copenhagen University Hospital-Hvidovre. Maximum three days before the procedure, blood samples will be taken to check BAC-test, thrombocytes, INR and haemoglobin. The participant must be fasting 4 hours before the procedure but can take his/her usual medication except for any blood-thinning medicines, which will be paused according to clinical guidelines. If any contraindications for the biopsy is found, the participant will be excluded from this part of the study. Two individual pathologists make the histological description after a standardised histological scoring system (47). Excess liver tissue from the biopsies will be used for transcriptomics, proteomics and metabolomics. The biopsy will be taken before and after the intervention. A liver biopsy is not mandatory for participation in the study.

4. STATISTICAL CONSIDERATIONS

No exercise intervention studies have been performed in patients with CHB. Using studies with similar exercise intervention but performed in NAFLD patients, we assume a standard deviation of 3.0 % and no change in the control group. An 80 % chance of detecting a -2.8 % between-group change in liver IHL with a one-sided 0.05 significance require n=15 in each group (27,26). Data will be analysed per protocol, and any drop-out will lead to the inclusion of a new study participant.

5. PATIENTS

Patients will be recruited at the Department of Infectious Diseases, Copenhagen University Hospital – Hvidovre and at the Department of Infectious Diseases, Copenhagen University Hospital - Rigshospitalet, at a regular outpatient visit. Recruitment posters will be posted online (see appendix 3) and patients will be able to contact the principal investigator if they are interested in being a part of the study.

Criteria of inclusion:

- CHB defined by HBsAg positive >6 months
- Positive HBV-DNA
- Age >30
- Hepatic steatosis diagnosed by Controlled Attenuated Parameter (CAP) >250 assessed by Transient Elastography

Criteria of exclusion:

- HIV, HCV, HDV-co infection
- Primary biliary cholangitis
- Wilsons Disease
- Autoimmune hepatitis
- Hepatocellular carcinoma
- Antiviral medication
- Steatogenic medication (systemic corticosteroids, amiodarone, tamoxifen, valproic acid, and methotrexate)
- Average alcohol intake >30 g for men and >20 g for women pr. day
- Unable to understand and read written information for participants written consent
- Pregnancy

6: RISKS, SIDE EFFECTS AND LONG- AND SHORT-TERM DISADVANTAGES

Adverse events will be reported annually. In case of a severe adverse event, the study's primary investigator will report this to the Regional Committees for Region Hovedstaden within seven days of notice. Suppose any situation occurs during the trial, which will be of relevance for the study participants safety or the execution of the trial. In that case, the Regional Committees for the Region Hovedstaden will be informed immediately.

Aerobic exercise training: The participants will be supervised during the exercise training and attended to by a medical doctor if they experience any signs of discomfort.

Blood samples: The blood samples will be obtained after sufficient hand hygiene and disinfection of the skin; sterile single-use needles will be used to reduce the risk of infection. An amount of maximum 400mL will be drawn from the participants. This amount will be a small amount of blood compared to the total blood volume and will not have any physical consequences for the participant. The prick of the needle can cause a little discomfort, and a small bruise might occur afterwards.

Continuous glucose monitoring: The method is safe and routinely used by patients with diabetes to monitor the blood glucose level continuously. A tiny electrode (glucose sensor) will be inserted into the subcutaneous tissue at the upper arm. To this end, there is a small risk of infection, and the study participants will be informed and instructed to get medical attention in case of symptoms or signs of infection.

Hormone infusion: Infusion of somatostatin in combination with glucagon infusion is safe. There is a minor risk of infection or hematoma due to bloodlines being placed. All researchers are experts in these procedures, so the risks are minimal.

Oral glucose tolerance test: The participants might feel nausea and discomfort after ingesting the glucose solution. Otherwise, this method is considered safe without side effects and is widely used in clinical practice.

DXA scan: The DXA scan will expose the patients to a minimal amount of radiation, varying between 3-30 µSv (microSievert) pr. scan, comparable to <1 week of background radiation. Considering the benefit of the expected results, the small amount of radiation is considered acceptable.

Transient Elastography: The examination is non-invasive and does not put the patients at any risk nor entails side effects or discomfort for the participants. The 5 hours fast prior to the procedure is considered acceptable.

MRS Liver and MRI visceral fat: The proton magnetic resonance spectroscopy and magnetic resonance imaging scan of the liver and visceral fat does not expose the participants for radiations but may be experienced to be long-lasting and to some claustrophobic. This is addressed by informing the patients thoroughly before the scans and using standard screening for metal objects; if a patient needs a sedative prior to the scan, a tablet of alprazolam 1 mg can be given to the patient 1 hour before the scan. A medical doctor will prescribe this medication and ensure there and no contraindications for this. Some people are sensitive to the contrast agent and may develop an allergic reaction. They will be attended by a medical doctor if developing an allergic reaction. The contrast used is considered safe and is approved by the European Medical Association (EMA).

Liver Biopsy: Percutaneous liver biopsy is an invasive procedure with known risks. The participants will be informed both written and orally about the procedure and the associated risks. The participants are informed that it is not compulsory to undergo a biopsy to participate in the study. After informed consent, the liver biopsy will be made and have the participant admitted to the Department of Infectious Diseases at Copenhagen University Hospital- Hvidovre. Study participants with contraindications to a liver biopsy, e.g. bleeding complications, will not undergo the procedure. Any blood-thinning medication will be paused according to clinical guidelines. A syringe which is 1,2 mm, will be used to take out approximately 34,5 mg of liver tissue. The department instructions for planned liver biopsy will be followed. After the procedure, the participant will be observed for at least 5 hours. Discomfort at the site of injection or in the right shoulder occurs in 1 out of 4 patients. The procedure is connected to a risk of bleeding. Severe bleeding is rare (occurring in 1/2500), with a need for hospitalisation and potential blood transfusion or operation(48). Most of the bleeding complications occur 2-4 hours after the procedure is done, and the participant is observed at the hospital, but there is a risk of bleeding up to a week after the procedure. The study participant will be informed about avoiding physical activity three days after discharge and are advised to contact a doctor if they experience increasing pain, bleeding for the insertion site or any other form of discomfort.

7: EXTRACTION OF BIOLOGICAL MATERIAL

How much and what: Blood will be drawn from the study participants 5 times. Before the exercise intervention, after four weeks, after eight weeks, and after the study intervention, during follow-up, blood will be drawn 6 and 12 months after the intervention. The maximum amount of blood drawn will be 400mL. Liver biopsies will be collected before and after the intervention. At each collection, two samples of a maximum of 34,5 mg will be taken. This will include a total of a maximum of 138 mg liver tissue.

For what purpose: Some of the blood will be analysed immediately for general tests like fasting glucose, fasting insulin, lipid status, HbA1c, haemoglobin, leucocytes and differential count, thrombocytes, CRP, ALT, basic phosphatase, LDH, bilirubin, INR, creatinine, carbamide, Na+, K+, creatine kinase, TSH. Other samples will be saved for analysis after the final participant has finished the study period. The blood kept for further analysis will be kept in a research biobank at -80 ^{o}c at Centre for Physical Research Activity, Copenhagen University Hospital -Rigshospitalet. These blood samples will be analysed for cytokines and hepatokines. Liver biopsies will be used for the histological description of the liver tissue. Any leftover tissue will be kept in a research biobank at the Department of Infectious Diseases, at Copenhagen University Hospital,

Hvidovre under the regulation by the Danish Data Protection Regulation (databeskyttelsesforordningen) and the Danish Data Protection Law (databeskyttelsesloven).

Biobank for future research: When all tests are finalised, any excess blood or plasma and liver tissue will be stored in the research biobank at -80°c until the study is finished (July 1st. 2023). After the study period, the excess samples of blood or plasma or liver tissue will be moved to a research biobank for future research, where they will be anonymised and stored further ten years after the end of the study (July 1st 2033). The biobank for future research will be under the regulation by the Danish Data Protection Regulation (databeskyttelsesforordningen) and the Danish Data Protection Law (databeskyttelsesloven). Suppose any later studies would like to use the biological material. In that case, this will only occur following approval by the Danish Data Protection Regulation (databeskyttelsesforordningen) and the Danish Data Protection Law (databeskyttelsesloven).

After informed consent, the study participants will also be asked if they will allow us to withdraw extra blood for a biobank for future research in a separate participant information document. The biobank for future unspecified research will be under the regulations and acceptance of the Danish Data Protection Regulation (databeskyttelsesforordningen) and the Danish Data Protection Law (databeskyttelsesloven). The blood stored in the biobank for future research will be used for future specified research if approved by the Regional Committees for the Region Hovedstaden and the study participants. Sometimes the Regional Committees can give allowance to do research without the study participants consent. Participants can decline to donate blood for the biobank for future unspecified research without any consequences. Study participants can at any time request to have their biological material destroyed. Such demand will cause immediate destruction.

Responsibility and access: Principal investigator Sofie Dikeledi Kold Jespersen and primary supervisor Nina Weis will have full access to the material in this period and be responsible for destroying all material after July 1st 2033. The biological material will not leave Denmark. All regulations by the Danish Data Protection Regulation (databeskyttelsesforordningen) and the Danish Data Protection Law (databeskyttelsesloven) are kept.

8: INFORMATION FROM PATIENTS JOURNALS

Patient journals will be accessed to screen CHB eligible candidates in the outpatient clinic at the Department of Infectious Diseases, Copenhagen University Hospital – Hvidovre and at the Department of Infectious Diseases, Copenhagen University Hospital – Rigshospitalet. The screening will be including the in- and exclusion criteria and will be passed on to the principal investigator. After informed consent to

participation in the project, patient demography, patients previous HBV treatment, fibroscan values, previous liver biopsies, HBV related blood samples, and comorbidities will be extracted from the journal. The information will be used to identify potential eligible candidates for study participation and when obtaining informed consent, the information will be used in the research project to describe the health information of the participant group and for statistical analysis.
The patients will be informed that the investigators, sponsor and sponsors representative and any potential control authority may have direct access to collect information in patient files including electronic patient file to find information about the study participants health conditions, which are necessary to complete the research project and for control purposes, including self-control, quality control, and monitoring, which the investigator and control authority are obliged to do.

9: PROCESSING OF PERSONAL DATA IN THE PROJECT

All patient-related information obtained during the study will be handled by the Danish Data Protection Regulation (databeskyttelsesforordningen) and the Danish Data Protection Law (databeskyttelsesloven). The blood samples will be registered from the hospital blood sample portal (Labka), and para-clinical observations will be obtained through "Sundhedsportalen". No personal information will be transferred abroad. Personally, identifiable data will be stored in a password secured web-based clinical trial management system EasyTrial database, which the Danish Data Protection Board has approved under Region H.

10. ECONOMY

Primary initiators are Professor, MD, PhD Nina Weis, Clinical Associate Professor, MD, PhD Rikke Krogh-Madsen and MD, PhD Student Sofie Kold Jespersen. The study collaborates between the Department of Infectious Diseases, Copenhagen University Hospital - Hvidovre, Centre for Physical Activity Research (CFAS), Copenhagen University Hospital – Rigshospitalet and the Department of Infectious Diseases, Copenhagen University Hospital - Rigshospitalet. The project is currently supported financially by CFAS with approximately 1.000.000 DKK for salary for PhD student Sofie Dikeledi Kold Jespersen and a project nurse, hormones, blood sample analysis, DXA-scans and exercise facilities, and 100.000 DKK from the Beckett Foundation for paraclinical analysis, and with 250.000 DKK from Holms Mindelegat for salary for PhD student Sofie Jespersen. The money from the Beckett Foundation and Holms Mindelegat is administered in a research account at Copenhagen University Hospital - Hvidovre. Further funding has been applied, and the Regional Committees for the Region Hovedstaden and the study participants will be informed when funding has been obtained.

None of the clinical investigators has financial affiliation to financial supporters or other stakeholders in this trial. There are no commercial interests in the project.

11. ALLOWANCES

Patients who will not be doing the exercise intervention will be offered a 3 months free membership to a gym like Fitness World (price 1000 DKK), after the have finished the study. Apart from this, patients will not be offered any financial remuneration for participation in the study. If a patient is financially challenged and unable to participate in the study due to transport expenses, the patients can receive refunds for documented transport expenses. Parking fees will be covered when parking at Rigshospitalet's parking lot.

12. RECRUITMENT OF PATIENTS WITH INFORMED CONSENT

The patients will be recruited through the outpatient clinic at the Department of Infectious Diseases, Copenhagen University Hospital – Hvidovre, at the Department of Infectious Diseases, Copenhagen University Hospital – Rigshospitalet, and through recruitment postings (see appendix 3). Eligible participants will be identified by the study investigator, who will contact the clinical staff treating the patient, whom will request, if the patient will accept to be informed about the study by the study investigator. Or the clinical staff treating the patient will ask, if the study participant will accept that they give the patients contact information to the study investigator, who can then contact the patient by phone or email and inform about the study. The recruitment postings will be posted in media: newspapers, (Politiken, Berlingske, BT, Information, Metro Express), through patient associations (Leverforeningen) at social media (Facebook, Twitter and LinkedIn), and by posters, and flyers in local areas and hospital departments. Interested persons who have seen the recruitment postings can then contact the project investigators by either email or by phone. When including patients, the patients will receive a written and oral presentation of the study in a closed environment without disturbances and the option for asking in-depth questions. The information will be given by the principal investigator. The study participants will be given time – up to 2 weeks- to consider if they are willing to participate. If requested, an assessor can participate and ask questions to the person, including the study participants. Inclusion will require written consent from study participants before enrolment in the study. The written consent will be stored at Centre for Physical Research Activity, Copenhagen University Hospital -Rigshospitalet, Blegdamsvej 9, 2100 Kbh Ø, behind two locked doors from public accessibility and not together with study participant files.

13. PUBLISHING OF STUDY RESULTS

The study results will be published as a scientific article in a peer-reviewed international journal and presented at national and international conferences. All results, positive, negative and inconclusive, will be published. There are no commercial interests preventing publication. Authorship will be acknowledged by use of the Vancouver criteria.

14 ETHICS IN SCIENCE:

Participants in the study will by participation in the entire examination programme, undergo the following tests: Clinical examination (including weight, height, blood pressure, EKG, waist circumference), blood samples, continuous glucose monitoring, physical activity monitoring, infusion of somatostatin and glucagon, oral glucose tolerance test, DEXA scan, transient elastography, MRS and MRI scans, liver biopsy. Risks and side effects are described in section 6. The participants can participate in the study without having a liver biopsy performed.

Any clinically relevant finding from the investigations mentioned above will be referred to further examination and treatment if indicated. Participation in the study will not influence medical treatment or care, and the patients will be followed as usual in the outpatient clinic at the Department of Infectious Diseases. At any point of participation in the study, the participants can declare a withdrawal and will be taken out of the study and not undergo follow-up if this is chosen.

We believe this study is essential to contribute to the understanding of the effects of comorbidity in chronic hepatitis B patients to improve health, quality of life, and life expectancy. To address the effects of comorbidity, we must increase our knowledge of how inflammatory and metabolic pathways are affected when patients are infected with the hepatitis B virus. The study will be requiring much time from the included patients. They will be using a lot of their time doing exercise training and have long test days. However, we believe the benefits of aerobic exercise training on general health will outweigh this. It is thus, to our understanding, ethically acceptable to complete this study.

15. INFORMATION AND COMPENSATION

All patients will be covered by The Danish Patient Insurance Association for any injury that may occur as a direct consequence of study-related procedures.
