# Supplementary material for: Effect of high-intensity interval training in patients with chronic hepatitis B and hepatic steatosis: A randomised controlled trial
Source: PLoS One. 2026 Jun 23;21(6):e0351547. doi: 10.1371/journal.pone.0351547 (PMC13289930; doi:10.1371/journal.pone.0351547)
Supplement: Attachment — Submitted filename: Response to editor.docx [file pone.0351547.s005.docx]

Re: **Effect of high-intensity interval training in patients with chronic hepatitis B and hepatic steatosis: a randomised controlled trial**

To the Academic Editor

Surya Prakash Bhatt, Ph.D

PLOS ONE

My co-authors and I thank you for considering our manuscript for publication in PLOS ONE. We are grateful for the comments from the Academic Editor, which we have followed and revised the manuscript accordingly. Please find enclosed a revised clean version of the manuscript and a version with revisions marked in tracked changes. We hope that the revised manuscript is satisfactory for publication. Thank you again for your interest in publishing our paper in PLOS ONE.

Yours sincerely

Sofie Dikeledi Kold Jespersen, MD, Ph.D

Reply to Academic Editor comments (author reply in blue)

Final Comments: Due to multiple critical issues—particularly underpowering, flawed inclusion criteria, lack of dietary control, and weak statistical rigor—this study cannot support its primary conclusions. If revised, it should be framed explicitly as a feasibility or pilot trial with exploratory outcomes only.
Response: Thank you for this thorough and thoughtful assessment. We acknowledge the editor’s concerns regarding statistical power, inclusion criteria, and the interpretation of the findings, and we have revised the manuscript to ensure a more cautious interpretation of the findings.

Importantly, the study was designed, prospectively registered, and accompanied by a published protocol as a randomised controlled trial (PMID: 37312098 and ClinicalTrial.gov registration number NCT05265026), and not as a pilot or feasibility study. The original intent was therefore to conduct a definitive RCT. However, due to slower-than-anticipated recruitment, the trial was prematurely discontinued, resulting in a smaller final sample size and limited statistical power.

We fully agree that this limitation must result in a cautious interpretation of the results. Accordingly, we have explicitly stated that the trial was discontinued early due to recruitment challenges and that all analyses should be regarded as exploratory and hypothesis-generating rather than confirmatory. This clarification has been added to the Abstract, Discussion, and Conclusions.

While the reduced sample size precludes definitive conclusions regarding efficacy, we believe that transparent reporting of the trial as conducted—without retrospectively reclassifying it as a pilot study—represents best practice and allows the findings to be interpreted appropriately within their methodological constraints.

We hope that the following revisions will allow the manuscript to be considered for publication.

1.Major Methodological Flaws
1.1 Inadequate Sample Size & Power
• The study was powered for 28 subjects but included only 14 completers. The under-recruitment renders the study severely underpowered, especially for detecting small-to-moderate effects in liver fat fraction.
• Given the non-significant p-values and wide confidence intervals, any inference about effectiveness is unjustified. The conclusion should be reframed as exploratory.

Response: We acknowledge this important limitation and agree with the editor’s assessment. The reduced sample size indeed limited the statistical power of our study, particularly for detecting small-to-moderate effects in liver fat fraction. We have revised the manuscript to explicitly frame the findings as exploratory and rephrased the conclusions to reflect the underpowered nature of the study.

Change in manuscript:

***Abstract:***

*Background & Aims:*

*Having both chronic hepatitis B and hepatic steatosis increases the risk of liver-related morbidity and mortality. We aimed to investigate if high-intensity interval training could decrease the liver fat-fraction and improve liver status, body composition, lipid- and glucose metabolism, and blood pressure in patients with chronic hepatitis B and hepatic steatosis.*

*Methods*

*In a randomised, controlled trial, patients with chronic hepatitis B and hepatic steatosis were randomised 1:1 to high-intensity interval training over 12 weeks or no intervention. The primary outcome was reduction in liver fat-fraction (≥ 2.8 %), assessed by magnetic resonance imaging. Secondary outcomes were body composition, indices of glucose metabolism, blood lipids, blood pressure, alanine aminotransferase, physical fitness assessed by maximal oxygen consumption (VO2max), and self-assessed health. The trial was discontinued before reaching the planned sample size due to slow recruitment.*

*Results*

*Nineteen patients were included, and 14 completed the trial (seven per group). Changes in liver fat-fraction showed a between-group difference of -2.03 % [95 % CI: -5.5 to 1.4; p=0.22]. Exercising patients improved VO2max by 5.6 mL/kg/min [1.7 to 9.5; p<0.05] and emotional well-being by 18.07 points [6.6 to 29.6; p<0.05]. The exercise intervention did not affect total body fat -0.35 % [-2.8 to 2.1] or lean body mass 0.6 kg [-0.7 to 1.9]. Changes in glucose tolerance, insulin secretion, Matsuda index -0.89 [-2.5 to 0.5; p=0.18], cholesterol 0.29 mmol/L [-0.3 to 0.9; p=0.32] and systolic blood pressure 6.36 mmHg [-6.4 to 19.1; p=0.29] did not differ significantly between groups.*

*Conclusions*

*We did not observe a significant effect of high-intensity interval training on liver fat-fraction in patients with chronic hepatitis B and hepatic steatosis. Due to premature discontinuation and the resulting limited sample size, the study was underpowered, and the findings should be interpreted as exploratory. Nevertheless, the intervention improved physical fitness and emotional well-being.*

***“Conclusion***

*This study, the first to investigate the effect of high-intensity interval training on hepatic steatosis in patients with CHB, is inconclusive due to premature discontinuation and the resulting smaller-than-planned sample size, leading to insufficient statistical power. Consequently, no definitive conclusions can be drawn regarding the effect of HIIT on the primary endpoint on liver fat-fraction, and on secondary outcomes, such as body composition, lipid and glucose metabolism, and blood pressure in patients with CHB. The findings should therefore be interpreted as exploratory. However, we found that the intervention was well-tolerated, with no safety concerns identified, and improved cardiorespiratory fitness by improving VO2max and self-reported emotional well-being.“*

1.2 Poor Screening Specificity
• CAP >250 dB/m was used to define hepatic steatosis; however, 42% of patients had <5% fat fraction on MRI-PDFF, the actual primary outcome metric. This undermines internal validity.
• The authors themselves suggest >300 dB/m may be more appropriate — this should have been anticipated or at least triggered a mid-study protocol amendment.

Response: We appreciate the editor’s observation. The CAP threshold (>250 dB/m) was specified in the original study protocol based on the best available evidence at the time of study design, most notably the meta-analysis by Karlas et al. (PMID: 28039099), which identified a cut-off around 246 dB/m for the detection of hepatic steatosis. At the time the study was initiated and conducted, there was no established consensus supporting a higher CAP threshold (e.g., >300 dB/m), nor was such a cut-off robustly validated against MRI-PDFF as a reference standard.

Importantly, MRI-PDFF—the primary outcome—was obtained only at study completion for logistical and methodological reasons. Consequently, the observed discrepancy between CAP-based inclusion criteria and MRI-PDFF–defined steatosis could not be identified during the conduct of the study and therefore could not reasonably trigger a protocol amendment or ethical addendum during enrollment.

In retrospect, and based on the present findings, a higher CAP threshold may indeed provide improved specificity when MRI-PDFF is used as the reference standard. This limitation has been explicitly acknowledged in the manuscript, and we agree that future studies should consider higher CAP cut-offs or incorporate baseline MRI-PDFF measurements to better align inclusion criteria with the primary outcome.

Change in manuscript: (under Strengths and Limitations)

*“In retrospect, the present results suggest that a higher CAP threshold (e.g., >300 dB/m) may provide improved specificity when MRI-PDFF is used as the reference standard. Future studies should consider using the higher suggested CAP cut-offs or incorporating baseline MRI-PDFF measurements to better align eligibility criteria with the primary outcome. Future studies should consider applying higher CAP cut-offs or combining CAP with baseline MRI-PDFF to better align inclusion criteria with the primary outcome.“*

1.3 Absence of Diet Control
• No dietary monitoring or standardization is a critical omission in a metabolic intervention study.
• Diet is a major confounder in hepatic steatosis studies, and the authors admit that at least two participants had major dietary changes.

Response: We acknowledge the editor’s concern and agree that dietary monitoring would have strengthened the study. However, our intention was to isolate the effects of the exercise intervention without imposing dietary restrictions, thereby reflecting real-world conditions. The two cases of major dietary changes were unforeseen and resulted from illness and lifestyle decisions, despite our requirement that participants maintain stable dietary habits throughout the study. This issue has been identified as an important limitation and has been emphasized in the revised manuscript, including that future studies should incorporate dietary monitoring.

Change in manuscript: (under Strengths and Limitations)

*“Another limitation is the lack of systematic diet control and monitoring during the intervention. A change in diet has been shown to affect the liver fat-fraction and visceral adiposity greatly (38). Although participants were instructed to maintain stable dietary habits throughout the study at least two patients in this study had major changes in food intake, which were not systematically or quantitively registered. These unanticipated changes, related to illness and religious lifestyle (Ramadan), may have influenced the primary and secondary outcomes and thereby reduced the ability to attribute observed effects solely to the exercise intervention. While the study was designed to examine the effects of exercise under real-world conditions without dietary restrictions, future studies would benefit from incorporating dietary monitoring or control to minimize confounding and improve interpretability.”*

1.4 Per-Protocol Bias and ITT Limitations
• Although per-protocol and ITT analyses are reported, the small sample size and lack of blinding make per-protocol results unreliable.
• The one subject with questionable adherence (Patient 5) was retained despite failure to meet HRmax goals. This introduces adherence bias.

Response: We appreciate the editor’s point. While the primary outcome was indeed assessed in a blinded manner, we acknowledge that secondary outcomes were not blinded, which may limit the reliability of those results. In addition, the small sample size and inclusion of Patient 5, who did not consistently achieve the prescribed HRmax target, may have introduced adherence-related bias. In the revised discussion, these limitations are now more clearly described.

Change in manuscript: (under Strengths and Limitations)

*“Although both intention-to-treat and per-protocol analyses were performed, the small sample size limits their robustness, and the per-protocol findings in particular should be interpreted with caution. While the primary outcome was assessed in a blinded manner, secondary outcomes were not blinded, which may have introduced measurement bias. In addition, one participant demonstrated suboptimal adherence to the prescribed training intensity yet was retained in the analyses for transparency, which may have contributed to adherence-related bias. Together, these factors further limit the interpretability and generalizability of the findings.”*

2. Statistical and Analytical Issues

2.1 Multiple Hypothesis Testing Without Correction
• A wide array of secondary outcomes was tested without adjustment for multiplicity. This raises the likelihood of Type I errors.
• The significant emotional well-being improvement (p < 0.05) could easily be a false positive. Use FDR or Bonferroni adjustment, or qualify results as exploratory.

Response: We agree with the editor’s comment regarding the risk of inflated Type I error due to multiple testing of secondary outcomes. In the revised manuscript, p-values for all secondary outcomes have been adjusted using Bonferroni correction. The effects observed for Emotional well-being and Improvement of cardiorespiratory fitness remain statistically significant after adjustment. All results presented without correction are now explicitly described as exploratory and hypothesis-generating.

2.2 Overinterpretation of Non-Significant Trends
• Several non-significant findings (e.g., liver fat reduction of -2.03%, p = 0.22) are framed too optimistically.
• Statements like “trend toward benefit” lack statistical support and should be removed or reworded to reflect the absence of evidence.

Response: We acknowledge this concern and agree that non-significant findings should not be overstated. The discussion and conclusion sections have been revised to ensure the interpretation is appropriately cautious regarding non-significant trends.

2.3 Use of Confidence Intervals
• Many CIs are extremely wide (e.g., insulin AUC: -22101 to 13980), indicating poor precision. This should be acknowledged explicitly in limitations.

Response. We agree with the editor’s observation. We have explicitly acknowledged this limitation in the revised manuscript.

Change in manuscript: (under Strengths and Limitations)

*“This study is primarily limited by a small sample size and substantial heterogeneity among participants with respect to ethnicity, body weight, and liver fat fraction. Together, these factors led to wide confidence intervals, diminished statistical power, and an increased risk of type II error, thereby constraining the robustness of the conclusions.”*

3. Issues in Study Design and Execution
3.1 Lack of Blinding in Outcome Assessments
• Apart from MRI fat fraction, all secondary outcomes (e.g., VO2max, SF-36, BP) were unblinded. This introduces measurement bias and threatens validity.

Response: We acknowledge this limitation. While the primary outcome was assessed in a blinded manner, secondary outcomes were not. The Limitations section has been revised to more clearly acknowledge this limitation.

Change in manuscript: (under Strength and Limitations)

*“While the primary outcome was assessed in a blinded manner, secondary outcomes were not blinded, which may have introduced measurement bias. In addition, one participant demonstrated suboptimal adherence to the prescribed training intensity yet was retained in the analyses for transparency, which may have contributed to adherence-related bias. Together, these factors further limit the interpretability and generalizability of the findings.”*

3.2 Heterogeneous Baseline Fat Fraction
• Wide variability in baseline liver fat across groups (10.4% vs. 5.1%) suggests imbalance post-randomization. No stratified randomization or covariate adjustment was applied.

Response: We acknowledge the observed baseline imbalance in liver fat fraction between groups. This difference likely reflects random variation due to randomization and the small sample size; with the originally planned sample size less variability would have been expected. This limitation is noted in the revised manuscript and clarify its potential impact on the interpretation of results.

Change in manuscript: (under Strength and Limitations)

*“Another important limitation of this study concerns the specificity of the screening criterion used to define hepatic steatosis. A CAP threshold >250 dB/m was applied at inclusion; however, 42% of participants had a hepatic fat fraction < 5% when assessed by MRI-PDFF, the primary outcome measure. This mismatch likely reduced the study’s ability to detect intervention-related effects and may have contributed to the inconclusive findings.*

*….*

*MRI-PDFF assessments were performed only at the end of the intervention, and the discrepancy between CAP-based screening and MRI-PDFF outcomes could therefore not be identified during the study.“*

3.3 Missing Pre-Specified Outcomes
• The study originally planned liver biopsies for histological analysis but dropped this due to non-consent. While practical, this change significantly weakens the outcome depth, and should be transparently discussed under limitations with more emphasis.

Response: We acknowledge this limitation. Although liver biopsies were originally planned for histological analysis, they were not performed due to lack of participant consent. The Limitations section is now expanded to discuss how this change reduces the depth of outcome assessment.

Change in manuscript: (under Strength and Limitations)

*“The absence of liver biopsy data further highlights the limitation of liver steatosis assessment. Although biopsies were planned, most participants declined, and only one was performed, leaving out pathological assessment of steatosis, inflammation and fibrosis.”*

4. Interpretation and Reporting Concerns
4.1 Inadequate Discussion of Null Results
• The discussion does not address the possibility that HIIT may truly have no effect in CHB-associated steatosis — only that the sample was too small.
• The authors do not critically engage with the negative findings and their implications for pathophysiology or clinical translation.

Response: We acknowledge this concern and agree that the discussion should more fully address the possibility that HIIT may have no effect in CHB-associated steatosis. The Discussion and Conclusion sections have been expanded to further explore this issue.

4.2 Generalizability Issues Ignored
• Only 19 of 695 screened patients were enrolled (2.7%). This extreme attrition suggests selection bias and limits external validity.
• Most participants were middle-aged, urban, and likely motivated — the results are not generalizable to broader CHB populations, especially in LMICs.

We acknowledge the editor’s concern about the study's limited generalizability. The low enrollment rate relative to the number of screened patients, together with the demographic and clinical characteristics of the included participants, introduces a risk of selection bias and limits external validity.

To ensure transparency, we have clearly reported participant characteristics in Table 1 and provided a detailed flow diagram outlining screening, exclusions, and enrollment. We believe this allows readers to assess the context and applicability of the results appropriately.

Change in manuscript: (under Strength and Limitations)

*“Another consideration is that the study population primarily consisted of motivated, middle-aged patients receiving care in a Danish clinical setting, which may limit the generalizability of the findings. The results may therefore not be directly applicable to broader populations of patients with CHB, particularly in lower- and middle-income countries or in healthcare systems with different organizational structures. Nevertheless, the intervention was delivered within routine clinical care, providing pragmatic insight into its feasibility and effects in a real-world, high-income setting.”*

4.3 SF-36 Emotional Well-being Score Overstated
• The absolute difference of 18 points is statistically significant but may not be clinically meaningful. Authors must discuss this with reference to known MCIDs for SF-36.

We acknowledge this point. The clinical significance of the 18-point change in SF-36 emotional well-being should be interpreted with caution. Accordingly, the Discussion in the Limitations section has been adjusted to reflect this consideration in more detail.

Change in manuscript: (under Discussion)

*“Our study shows that the self-assessed emotional well-being was increased in the exercise group. While the between-group difference of 18 points was statistically significant, the minimally important difference (MID) for SF-36 domains in chronic diseases is highly variable, ranging from 2–4 points in idiopathic pulmonary fibrosis (30) to 13–25 points in pulmonary arterial hypertension (31), and a 3-point change is often cited as a general threshold for summary scores (32)”*

5. Minor comments:
5.1 Incomplete Reporting of Supplementary Material
• Supplementary Tables are frequently referenced but were not included in the manuscript provided.

Response: Thank you for noting this. We originally sent in the supplementary Materials. But we have now ensured that all referenced supplementary tables are included in the revised manuscript.

5.2 Typos and Formatting Errors
• Several errors (e.g., "Hospiutal" instead of "Hospital") and inconsistent units (e.g., glucose AUCs, unclear standard deviations).

Response: Thank you for your careful review. We have thoroughly checked the manuscript and corrected spelling errors as well as formatting issues, including inconsistent units (e.g., glucose AUC from mmol/L*min to mmol/L x min) and a better impression of Table 1 with a clearer vision of standard deviations vs. mean (range) and median (IQR).

5.3 Duplicated or Misreferenced Literature
• Some references appear duplicated (e.g., Hallsworth studies), and some are outdated given the 2025 submission date.
Response: We appreciate the editor’s observations. The duplication of the Hallsworth article (references 10 and 26) is now corrected. Regarding the potentially outdated references, we would appreciate any specific suggestions or recommendations for more recent literature that we may have overlooked.
