# Supplementary material for: Effect of high-intensity interval training in patients with chronic hepatitis B and hepatic steatosis: A randomised controlled trial
Source: PLoS One. 2026 Jun 23;21(6):e0351547. doi: 10.1371/journal.pone.0351547 (PMC13289930; doi:10.1371/journal.pone.0351547)
Supplement: Attachment — Submitted filename: Response to reviewers 13052026.docx [file pone.0351547.s006.docx]

**Effect of high-intensity interval training in patients with chronic hepatitis B and hepatic steatosis: a randomized controlled trial**

To the Reviewers and Academic Editor, Sophia Eugenia Martínez-Vázquez, PhD

Dear Reviewers and Academic Editor,

My co-authors and I sincerely thank you for considering and reviewing our manuscript for publication in PLOS ONE. We are very grateful for the thoughtful and constructive comments provided by both Reviewers and the Academic Editor.

We have carefully addressed all comments and revised the manuscript accordingly. Please find enclosed a clean revised version of the manuscript and a version with tracked changes highlighting the revisions. We hope that the revisions adequately address your concerns and that the manuscript is now suitable for publication.

Thank you again for your time and consideration.

Yours sincerely, on behalf of the authors

Sofie Jespersen, MD, PhD

Department of Infectious Diseases

Copenhagen University Hospital, Hvidovre

Denmark

**Reply to the Academic Editors Comments (Author reply in blue):**

Dear Authors
Thank you for the opportunity to revise your manuscript about "Effect of high-intensity interval training in patients with chronic hepatitis B and hepatic steatosis: a randomized controlled trial".

Reply: Thank you very much for taking the time to review and comment on our manuscript.

Some points called my attention:

1. Please explain the real difference of hepatic steatosis by chronic hepatitis B in terms of treatment, that is to say, why a clinical study was needed to demonstrate that the same recommendation of exercise for other origin of the liver steatosis need to be proved in CHB?

Reply: We agree that the rationale for conducting a clinical study specifically in patients with CHB and hepatic steatosis could be articulated more clearly. While lifestyle interventions, including exercise, are well established in the management of hepatic steatosis of metabolic origin (MASLD, previously terminology NAFLD, which was used when designing the study) with a recommendation of >150 min/week of moderate or 75 min/week of vigorous intensity activity( ref: European Association for the Study of the Liver (EASL); European Association for the Study of Diabetes (EASD); European Association for the Study of Obesity (EASO). EASL-EASD-EASO Clinical Practice Guidelines on the management of metabolic dysfunction-associated steatotic liver disease (MASLD). J Hepatol. 2024 Sep;81(3):492-542. doi: 10.1016/j.jhep.2024.04.031. Epub 2024 Jun 7. PMID: 38851997.), it cannot be assumed that these effects are directly transferable to patients with CHB.

The underlying pathophysiology of hepatic steatosis in CHB may differ from that of MASLD in several important respects. In CHB, viral factors, host immune response, and interactions between hepatitis B virus replication and hepatic lipid metabolism may influence both the development of steatosis and its response to interventions. Additionally, hepatic steatosis in CHB has been associated with altered disease progression, including potential effects on fibrosis progression and hepatocellular carcinoma risk, which may not mirror patterns seen in MASLD.

Furthermore, current clinical guidelines, including those from EASL concerning management of CHB (ref: European Association for the Study of the Liver. EASL Clinical Practice Guidelines on the management of hepatitis B virus infection. J Hepatol. 2025 Aug;83(2):502-583. doi: 10.1016/j.jhep.2025.03.018. Epub 2025 May 10. PMID: 40348683.), do not provide specific recommendations for managing patients with concomitant CHB and hepatic steatosis. As a result, there is a lack of evidence-based guidance on whether exercise interventions, such as high-intensity interval training, are comparably efficacious and safe in this population.

Therefore, we thought that a clinical study was needed to specifically investigate whether the beneficial effects of exercise observed in MASLD populations could be replicated in patients with CHB, and to better inform clinical management in this distinct and understudied group of patients.

We have clarified this rationale in the revised introduction.

Page 4-5, line 90-100: “Hepatic steatosis in patients with CHB may differ from steatosis of metabolic origin in terms of underlying pathophysiology. In CHB, viral factors and host–virus interactions may influence lipid metabolism and disease progression, raising uncertainty as to whether treatment strategies established for NAFLD can be directly applied to this population.

The European Association for the Study of the Liver (EASL) guidelines provide no guidance on how to manage patients with both CHB and hepatic steatosis (8). Previous similar exercise intervention studies in patients with NAFLD (without CHB) have shown decreased liver fat-fraction and improved peripheral insulin resistance and liver fatty acid metabolism (9–11). Therefore, because patients with CHB have been excluded from these studies, evidence for similar benefits in this group is lacking.”

2. About sample size, please state if it was not reached because the prevalence of the disease in your hospital, city or country; or other condition.

Reply: We believe that another reason for not reaching the target sample size was the relatively low prevalence of the condition in our country compared to other regions such as Asia and Africa, where the burden of disease is substantially higher. This inherently limited the pool of eligible patients available for inclusion. It is estimated that the prevalence of chronic hepatitis B in Denmark is approximately 14.000 persons which corresponds to a 0.3 % of the population (ref. Bollerup S et al. see below)

We have clarified this point in the revised manuscript with the following:

Page 26, line 454 to 457: “Due to slow recruitment, we decided to close the study before reaching the intended number of patients. This challenge is likely multifactorial. CHB has a relatively low prevalence in Denmark (approximately 0.3%) (ref. Bollerup S, Wessman M, Hansen JF, Nielsen S, Hay G, Cowan S, Krarup H, Omland L, Jepsen P, Weis N, Christensen PB. Increasing prevalence of chronic hepatitis B virus infection and low linkage to care in Denmark on 31 December 2016 - an update based on nationwide registers. Infect Dis (Lond). 2023 Jan;55(1):17-26. doi: 10.1080/23744235.2022.2125065. Epub 2022 Oct 11. PMID: 36221255.). ), which restricts the number of eligible patients available for inclusion compared to higher-prevalence regions. In addition, patient-related factors may have influenced recruitment.

3. Explain the reason that the initial elastography was not in fasting. It generates concerns about the real steatosis stage at the time of diagnosis and then in the change of stage during the study.

Reply: We agree that fasting conditions may influence elastography measurements and, consequently, the assessment of steatosis stage.

In our clinical setting, it was not feasible to ensure that all patients were fasting prior to the baseline elastography assessment, as participants were recruited during routine outpatient visits. However, all patients are regularly followed in the clinic and are advised to attend consultations in a fasting state, as elastography is performed routinely as part of their clinical care.

Importantly, this limitation applied uniformly at baseline and reflects real-world clinical practice. Nonetheless, we acknowledge that non-fasting status may have introduced variability in TE-based steatosis measurements at baseline, and we hope to have addressed this transparently in the manuscript.

We would also like to emphasize that MRI-PDFF, for which all patients were examined under fasting conditions, was the primary outcome measure for hepatic steatosis in this study. TE was used as part of the initial assessment; however, in retrospect, a stricter standardization of fasting conditions for baseline TE may have been preferable.

Here is the revision we made in the Methods section:

Page 8, line 162-164: “TE performed prior to inclusion was not consistently conducted under fasting conditions, however intended, as these assessments were carried out during routine outpatient visits where fasting could not be systematically ensured. “

**Reply to reviewers' comments (author reply in blue):**

Reviewer #1: GENERAL COMMENTS

Thank you for the opportunity to review manuscript PONE-D-25-33230R1 titled “Effect of high-intensity interval training in patients with chronic hepatitis B and hepatic
steatosis: a randomised controlled trial”. I note here that I have not previously reviewed this.

Despite the need to end this study before a sufficient sample size was reached, I think this is a timely, important, objective, and well-done study overall, The former notwithstanding, two broad concerns, included in my specific comments below, have to do with whether some of the participants were already exercising prior to enrollment as well as whether the investigative team accounted for dropouts when determining sample size requirements. Finally, some minor improvement in the writing of this manuscript in the English-language is needed.

Reply: Thank you very much for your positive and encouraging feedback. We appreciate your constructive comments, and we have addressed each point in the revised manuscript.

SPECIFIC COMMENTS

*Page 3 and 4 (Abstract) – A nicely done, honest, and objective abstract.

Reply: Thank you.

*Page 5, lines 109 through 119 (Materials and Methods, Participants and eligibility) – Did you not include as part of your inclusion criteria that participants were not currently engaged in a regular exercise program?

Reply: At inclusion, all participants were asked about their exercise habits, and none were engaged in regular high-intensity exercise. Some participants did have access to a gym, but they were not performing structured high-intensity exercise or attending on a weekly basis. Formally, being currently engaged in a regular exercise program was not an exclusion criterion; in retrospect, incorporating this criterion might have been preferable to further standardize baseline physical activity levels.

*Page 6, lines 122 through 128 (Materials and Methods, Intervention) – Please tell the reader if these exercise sessions were supervised, and if so, by who. As I read ahead to page 22, line 389, I see that the exercise sessions were supervised.

Reply: We have clarified in the revised manuscript that all exercise sessions were supervised. Specifically, we have added the following text to the Methods section:

Page 6, line 134-136: “The supervision of each exercise session was performed by personnel from the Centre of Physical Activity Research, including sports science students, physiotherapy students, and medical students.”

For additional detail on the intervention, we also refer to our previously published protocol, which provides a comprehensive description of the exercise program and its supervision. (ref: Jespersen S, Plomgaard P, Madsbad S, Hansen AE, Bandholm T, Pedersen BK, et al. Effect of aerobic exercise training on the fat fraction of the liver in persons with chronic hepatitis B and hepatic steatosis: Trial protocol for a randomized controlled intervention trial- The FitLiver study. Trials. 2023 Jun 13;24(1):398)

*Page 6, lines 132 through 142 (Materials and Methods, Outcomes, Liver fat-fraction) – Please provide data on the accuracy and precision of your MRI proton density fat-fraction assessment. The same for the assessment of your other outcomes, which in some cases would be validity and reliability data, e.g., SF-36.

Reply: We have clarified the accuracy and reliability of the outcome measures in the Methods section and added appropriate references.

Page 7, line 152-156: “MRI-PDFF is a well-validated, non-invasive method for quantifying liver fat, demonstrating high diagnostic accuracy and reproducibility. Meta-analytic data show excellent performance across steatosis grades, with areas under the curve (AUC) values ranging from 0.91 to 0.97 and high sensitivity and specificity, supporting its robustness for both detection and grading of hepatic steatosis . (ref: Azizi N, Naghibi H, Shakiba M, Morsali M, Zarei D, Abbastabar H, Ghanaati H. Evaluation of MRI proton density fat fraction in hepatic steatosis: a systematic review and meta-analysis. Eur Radiol. 2025 Apr;35(4):1794-1807. doi: 10.1007/s00330-024-11001-1. Epub 2024 Sep 10. PMID: 39254718.)”

And

Page 10, line 206-211: ”SF-36 is a well-validated and reliable instrument for assessing health-related quality of life (ref: Brazier JE, Harper R, Jones NM, O'Cathain A, Thomas KJ, Usherwood T, Westlake L. Validating the SF-36 health survey questionnaire: new outcome measure for primary care. BMJ. 1992 Jul 18;305(6846):160-4. doi: 10.1136/bmj.305.6846.160. PMID: 1285753; PMCID: PMC1883187.). The SF-36 has consistently demonstrated strong internal consistency, with Cronbach’s alpha coefficients typically exceeding 0.85, and good test–retest reliability, with intraclass correlation coefficients often above 0.70. Its validity and responsiveness to changes in health status have been well documented across diverse populations. (ref: Kwan YH, Fong WW, Lui NL, Yong ST, Cheung YB, Malhotra R, Østbye T, Thumboo J. Validity and reliability of the Short Form 36 Health Surveys (SF-36) among patients with spondyloarthritis in Singapore. Rheumatol Int. 2016 Dec;36(12):1759-1765. doi: 10.1007/s00296-016-3567-3. Epub 2016 Sep 23. PMID: 27664139. And Wu Q, Chen Y, Zhou Y, Zhang X, Huang Y, Liu R. Reliability, validity, and sensitivity of short-form 36 health survey (SF-36) in patients with sick sinus syndrome. Medicine (Baltimore). 2023 Jun 16;102(24):e33979. doi: 10.1097/MD.0000000000033979. PMID: 37327281; PMCID: PMC10270486.)”

*Page 9, line 194 through 199 (Materials and Methods, Randomization and blinding) – First, please tell the reader who was responsible for conducting the randomization. Second, rather than saying “a co-author” on line 196, state the author per the author order in the manuscript, for example, “The second author assessed…”

Reply: We have clarified the randomization process in the revised manuscript, specifying who was responsible and using author order rather than a general description under the section Randomization and blinding.

Page 10, line 215-223: “The randomization sequence was generated using Sealedenvelope.com with varying block sizes, as described in detail elsewhere (Jespersen S, Plomgaard P, Madsbad S, Hansen AE, Bandholm T, Pedersen BK, Ritz C, Weis N, Krogh-Madsen R. Effect of aerobic exercise training on the fat fraction of the liver in persons with chronic hepatitis B and hepatic steatosis: Trial protocol for a randomized controlled intervention trial- The FitLiver study. Trials. 2023 Jun 13;24(1):398. doi: 10.1186/s13063-023-07385-y. PMID: 37312098; PMCID: PMC10262472). The envelopes were prepared by an independent researcher not involved in participant enrollment or assessment. The first author (S. Jespersen) enrolled participants and opened the envelopes sequentially immediately after baseline assessments, assigning participants irreversibly to their group. Neither the patients nor the primary investigator were blinded. The third author (C.Durrer) assessed the primary outcome of liver fat-fraction blinded without prior involvement with participants or examinations, and the primary investigator assessed the secondary outcomes unblinded.“

*Page 10, lines 202 through 204 (Materials and Methods, Changes from the original protocol) – Unless part of the original protocol, it would seem appropriate to tell the reader that you stopped the trial early along with why the trial was stopped early.

Reply: Very good point. We have added the following sentence to the “Changes from the original protocol” section:

Page 11, line 228-229: “Additionally, the trial was stopped early before reaching the planned sample size due to slow recruitment.”

*Page 10, lines 215 through 218 (Materials and Methods, Sample size considerations) – In your protocol, was any plan made to account for dropouts when determining your initial sample size? If so, then please describe. If not, then please state that also. As we all know, dropouts in exercise studies are very common, especially for those with chronic conditions.

Reply: No specific plan was included in the original protocol to account for dropouts when determining the initial sample size, as our Center for Physical Activity Research has historically observed low dropout rates in supervised exercise studies. Nevertheless, we aimed to include 30 participants in total, slightly exceeding the 28 participants required by the sample size calculation for the primary outcome, thereby providing a small buffer against potential dropouts. We have added the following sentence to the “Sample size considerations” section:

Page 11, line 243-245: “we aimed to include 30 participants in total, slightly exceeding the calculated requirement, providing a small buffer against potential dropouts.“

*Pages 10 and 11, lines 221 through 241 (Materials and Methods, Statistical analysis) – First, for your baseline characteristics (lines 221 through 223), suggest that you test for between-group differences and tell the reader what test(s) were used. Second, on lines 232 and 233, there are multiple different ways to conduct intention-to-treat analysis. Please describe the one you used, for example, last observation carried forward.

Reply: Thank you for this comment. Firstly, we have chosen not to report p-values for baseline characteristics, in line with CONSORT recommendations for randomized controlled trials. Proper randomization should ensure that any baseline differences are due to chance, making statistical testing unnecessary and potentially misleading. However, we understand your suggestion, particularly given the small sample size. We carefully reviewed the baseline characteristics and did not identify any clinically relevant imbalances between the groups. In acknowledgement of your comment, we also performed statistical comparisons using Student’s t-test, Mann–Whitney U test, and Fisher’s exact test where appropriate, which confirmed that there were no statistically significant between-group differences at baseline.

Secondly, the ITT analysis was conducted using a linear mixed-effects model including all randomized participants with available data, without imputation or last observation carried forward, under a missing-at-random assumption using maximum likelihood estimation. We have added the following sentence to the Statistical analysis section:

Page 20, line 263-266: “The ITT analysis included all randomized participants with available data and was conducted without imputation of missing data or use of last observation carried forward, relying on maximum likelihood estimation under the assumption that data were missing at random.”

*Pages 20 through 24, lines 336 through 450 (Discussion) – For ease of reading, suggest you partition your Discussion into the following subsections and address: (1) Overall findings, (2) Implications for research, (3) Implications for practice, (4) Strengths and potential limitations. Also, while a secondary outcome in your study, it may be worth mentioning, with a citation or two, that VO2 max is an important clinical marker, regardless of the population studied.

Reply: Thank you for this helpful suggestion. We have revised the Discussion section to include the recommended subsections. In addition, we have expanded the Discussion section to better explain the relevance of our findings. Specifically, we have emphasized the clinical relevance of the observed improvement in cardiorespiratory fitness:

Page 24, line 425-431: “The improvement in cardiorespiratory fitness found in the study, as assessed by VO₂max, is an important finding since it is a well-established independent predictor of cardiovascular and all-cause mortality and is widely used for risk stratification and clinical decision-making across a range of conditions (Ref: Abdalla HM, Dreher L, VanDolah H, Bacon A, El-Nayir M, Abdelnabi M, et al. VO2 Max in Clinical Cardiology: Clinical Applications, Evidence Gaps, and Future Directions. Curr Cardiol Rep. 2025 Sep 6;27(1):130. doi:10.1007/s11886-025-02289-6 PubMed PMID: 40913731). Additionally, higher baseline cardiorespiratory fitness has been associated with reductions in liver fat and may contribute to the resolution of hepatic steatosis independent of adiposity (Ref: Kantartzis K, Thamer C, Peter A, Machann J, Schick F, Schraml C, et al. High cardiorespiratory fitness is an independent predictor of the reduction in liver fat during a lifestyle intervention in non-alcoholic fatty liver disease. Gut. 2009 Sep;58(9):1281–8. doi:10.1136/gut.2008.151977 PubMed PMID: 19074179), supporting the clinical relevance of the observed improvements in VO₂max in this study.”

We have also expanded the section on implications for future research:

Page 24, line 410-417: “This study highlights several important considerations for future research. Accurate identification of hepatic steatosis at baseline is critical, and the observed discrepancy between CAP and MRI-PDFF suggests that more stringent cut-offs or combined diagnostic approaches may be warranted. Future studies should aim for larger sample sizes to ensure adequate statistical power and consider multicenter designs, particularly in regions with higher CHB prevalence. In addition, incorporating baseline MRI-PDFF measurements and more systematic dietary monitoring would improve internal validity and interpretability.”

*Pages 12 through 14 (Table 1) – As previously suggested, analyze for statistically significant differences in baseline characteristics between the exercise and control groups. This could be done by simply adding a column with a p-value.

Reply: As previously addressed, we chose not to report p-values for baseline characteristics in accordance with CONSORT recommendations. However, we carefully reviewed the baseline characteristics and did not identify any clinically relevant imbalances between the groups.

END OF REVIEW

Reviewer #2: General comments:

The reframing of the findings and the correction of the text accordingly are extremely welcome. The emphasis on effect size and 95% CI rather than post-hoc power is a correct approach. However, please clarify which estimation is being targeted.

Reply: We understand the reviewer’s point as a request to clarify the estimand, i.e., the specific treatment effect targeted by our analyses.

In the primary analysis, we estimated the between-group difference in change in liver fat-fraction from baseline to 12 weeks among participants meeting the predefined adherence criteria (per-protocol population). This was analysed using an ANCOVA model with follow-up liver fat-fraction as the outcome and baseline liver fat-fraction as a covariate, corresponding to a per-protocol efficacy estimand.

In addition, we have performed a secondary intention-to-treat analysis using linear mixed-effects models, including all randomized participants with available data, under the assumption of missing at random. This reflects the effect of treatment assignment in the randomized population.

Given the premature termination of the study and limited sample size, both estimates and their corresponding 95% confidence intervals are interpreted as exploratory.

We have rephrased the conclusion to the following:

Page 516, line 517-525: “This study is the first to investigate the effects of high-intensity interval training on hepatic steatosis in patients with CHB. Due to premature discontinuation and the resulting smaller-than-planned sample size, the study was underpowered, and the estimated effects on liver fat-fraction and secondary outcomes, such as body composition, lipid and glucose metabolism, and blood pressure should therefore be interpreted as exploratory rather than confirmatory. However, we found that the intervention was well-tolerated, with no safety concerns identified, and that it improved cardiorespiratory fitness by improving VO2max and self-reported emotional well-being. “

If MRI-PDFF was measured only at the end of the study, the change cannot be calculated. However, group differences can be reported at the endline. This approach risks bias if baseline imbalance exists. If a change has been reported, MRI-PDFF must have been measured at baseline as well. Therefore, the statement 'MRI-PDFF was only obtained at the end of the study' is inconsistent and should be corrected.

Reply: The MRI-PDFF scans were performed both before and after the intervention, allowing calculation of changes over time. The MRI-PDFF evaluation/assessments of liver fat fraction were performed only at the end of the intervention for analysis purposes, meaning that the analyses were conducted after completion of the study intervention.

We have corrected the text in the manuscript to clarify this under the Strengths and Limitations section:

Page 26, line 472- 478: “MRI-PDFF scans were performed at both baseline and at the end of the intervention, allowing assessment of change in liver fat-fraction over time. However, baseline MRI-PDFF was not used as an eligibility criterion; instead, inclusion was based on CAP or ultrasound findings, as specified in the protocol. Consequently, the discrepancy between CAP-based screening and MRI-PDFF-defined hepatic steatosis was only identified after completion of MRI-PDFF analyses and could not be accounted for during participant selection or eligibility assessment.”

It is positive from a transparency perspective that you stated PP/ITT analyses were performed and that participants with adhesion problems were excluded from the analyses. However, clarification may be needed regarding how ITT was implemented, the assumption of missing data, and the decision not to use LOCF. Furthermore, the pre-defined adhesion thresholds for PP analysis, how these thresholds are defined in the protocol, and how they are measured should be clarified.

Reply: Thank you for this comment. We have clarified the implementation of the ITT analysis in the revised manuscript. Specifically, we have added the following description to the Statistical analysis section:

Page 12, line 263-266: “The ITT analysis included all randomized participants with available data and was conducted without imputation of missing data or use of last observation carried forward, relying on maximum likelihood estimation under the assumption that data were missing at random.”

Regarding the PP analysis, the adherence thresholds were pre-defined in the study protocol (Ref: Jespersen S, Plomgaard P, Madsbad S, Hansen AE, Bandholm T, Pedersen BK, Ritz C, Weis N, Krogh-Madsen R. Effect of aerobic exercise training on the fat fraction of the liver in persons with chronic hepatitis B and hepatic steatosis: Trial protocol for a randomized controlled intervention trial- The FitLiver study. Trials. 2023 Jun 13;24(1):398. doi: 10.1186/s13063-023-07385-y. PMID: 37312098; PMCID: PMC10262472) and have now been explicitly described in the manuscript. We have added the following to the Statistical analysis section:

Page 12, line 254-257: “The adherence thresholds for the PP analysis were pre-defined in the study protocol. Participants were required to complete ≥70 % of the planned 36 exercise sessions and achieve ≥50 % of the prescribed HRmax during the sessions to be included in the PP analysis.”

In addition, we have clarified how adherence was measured in the Methods section under Intervention

Page 6, line 136-139: “Adherence was assessed and recorded by supervising trainers during each session using exercise logs and heart rate monitoring. For sessions with missing heart rate data, exercise duration was recorded, and the participant’s average training intensity from other sessions was used to estimate intensity.”

The authors applied a Bonferroni adjustment, and EWB and VO₂max remained statistically significant. For transparency, please specify the number of hypotheses included in the multiplicity adjustment and report, for each secondary endpoint, both the raw p‑values and the Bonferroni‑adjusted p‑values. Also indicate that tests were two‑sided and provide the corresponding α_adj (ex., 0.05/K).

Reply: Thank you for this comment. For transparency, we have now clarified in the manuscript that the Bonferroni correction was applied for 32 outcomes. The following legend is presented below Table 2:

Page 20, line 331-333: “Statistical significance was determined using a Bonferroni-adjusted alpha = 0.0016 (0.05/32), all tests were two-sided, and both raw and Bonferroni-adjusted p-values (calculated as raw p-value × 32, capped at 1) are reported for transparency.”

We have also added an extra column in Table 2 showing, for each secondary endpoint, both the raw p-values and the Bonferroni-adjusted p-values. To show that emotional well-being score and VO₂max remained statistically significant after adjustment.
