# Supplementary material for: Effect of high-intensity interval training in patients with chronic hepatitis B and hepatic steatosis: A randomised controlled trial
Source: PLoS One. 2026 Jun 23;21(6):e0351547. doi: 10.1371/journal.pone.0351547 (PMC13289930; doi:10.1371/journal.pone.0351547)
Supplement: Peer review history

# Editor report: Decision Letter 0

**DOI:** 10.1371/journal.pone.0351547.r001

**Authors:** Surya Prakash Bhatt

15 Jul 2025

PONE-D-25-33230

Effect of high-intensity interval training in patients with chronic hepatitis B and hepatic steatosis: a randomised controlled trial

PLOS ONE

Dear Dr. Jespersen,

Thank you for submitting your manuscript to PLOS ONE. After careful consideration, we feel that it has merit but does not fully meet PLOS ONE’s publication criteria as it currently stands. Therefore, we invite you to submit a revised version of the manuscript that addresses the points raised during the review process.

Please submit your revised manuscript by Aug 29 2025 11:59PM. If you will need more time than this to complete your revisions, please reply to this message or contact the journal office at plosone@plos.org. When you're ready to submit your revision, log on to https://www.editorialmanager.com/pone/ and select the 'Submissions Needing Revision' folder to locate your manuscript file.

Please include the following items when submitting your revised manuscript:

- A rebuttal letter that responds to each point raised by the academic editor and reviewer(s). You should upload this letter as a separate file labeled 'Response to Reviewers'.
- A marked-up copy of your manuscript that highlights changes made to the original version. You should upload this as a separate file labeled 'Revised Manuscript with Track Changes'.
- An unmarked version of your revised paper without tracked changes. You should upload this as a separate file labeled 'Manuscript'.

If you would like to make changes to your financial disclosure, please include your updated statement in your cover letter. Guidelines for resubmitting your figure files are available below the reviewer comments at the end of this letter.

If applicable, we recommend that you deposit your laboratory protocols in protocols.io to enhance the reproducibility of your results. Protocols.io assigns your protocol its own identifier (DOI) so that it can be cited independently in the future. For instructions see: https://journals.plos.org/plosone/s/submission-guidelines#loc-laboratory-protocols. Additionally, PLOS ONE offers an option for publishing peer-reviewed Lab Protocol articles, which describe protocols hosted on protocols.io. Read more information on sharing protocols at https://plos.org/protocols?utm_medium=editorial-email&utm_source=authorletters&utm_campaign=protocols.

We look forward to receiving your revised manuscript.

Kind regards,

Surya Prakash Bhatt, Ph.D

Academic Editor

PLOS ONE

Journal Requirements:

When submitting your revision, we need you to address these additional requirements.

1. Please ensure that your manuscript meets PLOS ONE's style requirements, including those for file naming. The PLOS ONE style templates can be found at

https://journals.plos.org/plosone/s/file?id=wjVg/PLOSOne_formatting_sample_main_body.pdf and

https://journals.plos.org/plosone/s/file?id=ba62/PLOSOne_formatting_sample_title_authors_affiliations.pdf

2. Thank you for stating the following in the Competing Interests section:

“Disclosure: Sten Madsbad declares; Advisory boards: AstraZeneca; Boehringer Ingelheim; Novo Nordisk; Sanofi, Abbott Lab, Bayer, Amgen.  Lecture fees: Novo Nordisk, MSD, E Lilly. Research Grant Recipient: Novo Nordisk; Novo Nordisk foundation, Boehringer Ingelheim. Grants were paid to the institution Hvidovre hospital, University of Copenhagen (no personal fee). None of the grants have any relation to the work presented in the paper.  Support for attending meetings and/or travel: Novo Nordisk, Boehringer-Ingelheim, Bayer. SMA has served as PI in relation to development of drugs for treatment of Type 2 diabetes and obesity in collaboration with Novo Nordisk and Bayer with funds paid to the institution where he is employed (no personal fee), and with no relation to the work reported in this paper.  Consultant for Netdoktor. All other authors declare no competing interests.”

Please confirm that this does not alter your adherence to all PLOS ONE policies on sharing data and materials, by including the following statement: "This does not alter our adherence to PLOS ONE policies on sharing data and materials.” (as detailed online in our guide for authors http://journals.plos.org/plosone/s/competing-interests). If there are restrictions on sharing of data and/or materials, please state these. Please note that we cannot proceed with consideration of your article until this information has been declared.

Please include your updated Competing Interests statement in your cover letter; we will change the online submission form on your behalf.

3. We note that you have indicated that there are restrictions to data sharing for this study. For studies involving human research participant data or other sensitive data, we encourage authors to share de-identified or anonymized data. However, when data cannot be publicly shared for ethical reasons, we allow authors to make their data sets available upon request. For information on unacceptable data access restrictions, please see http://journals.plos.org/plosone/s/data-availability#loc-unacceptable-data-access-restrictions.

Before we proceed with your manuscript, please address the following prompts:

a) If there are ethical or legal restrictions on sharing a de-identified data set, please explain them in detail (e.g., data contain potentially identifying or sensitive patient information, data are owned by a third-party organization, etc.) and who has imposed them (e.g., a Research Ethics Committee or Institutional Review Board, etc.). Please also provide contact information for a data access committee, ethics committee, or other institutional body to which data requests may be sent.

b) If there are no restrictions, please upload the minimal anonymized data set necessary to replicate your study findings to a stable, public repository and provide us with the relevant URLs, DOIs, or accession numbers. Please see http://www.bmj.com/content/340/bmj.c181.long for guidelines on how to de-identify and prepare clinical data for publication. For a list of recommended repositories, please see https://journals.plos.org/plosone/s/recommended-repositories. You also have the option of uploading the data as Supporting Information files, but we would recommend depositing data directly to a data repository if possible.

Please update your Data Availability statement in the submission form accordingly.

4. Please include captions for your Supporting Information files at the end of your manuscript, and update any in-text citations to match accordingly. Please see our Supporting Information guidelines for more information: http://journals.plos.org/plosone/s/supporting-information.

5. If the reviewer comments include a recommendation to cite specific previously published works, please review and evaluate these publications to determine whether they are relevant and should be cited. There is no requirement to cite these works unless the editor has indicated otherwise.

**Additional Editor Comments:**

1.Major Methodological Flaws

1.1 Inadequate Sample Size & Power

• The study was powered for 28 subjects but included only 14 completers. The under-recruitment renders the study severely underpowered, especially for detecting small-to-moderate effects in liver fat fraction.

• Given the non-significant p-values and wide confidence intervals, any inference about effectiveness is unjustified. The conclusion should be reframed as exploratory.

1.2 Poor Screening Specificity

• CAP >250 dB/m was used to define hepatic steatosis; however, 42% of patients had <5% fat fraction on MRI-PDFF, the actual primary outcome metric. This undermines internal validity.

• The authors themselves suggest >300 dB/m may be more appropriate — this should have been anticipated or at least triggered a mid-study protocol amendment.

1.3 Absence of Diet Control

• No dietary monitoring or standardization is a critical omission in a metabolic intervention study.

• Diet is a major confounder in hepatic steatosis studies, and the authors admit that at least two participants had major dietary changes.

1.4 Per-Protocol Bias and ITT Limitations

• Although per-protocol and ITT analyses are reported, the small sample size and lack of blinding make per-protocol results unreliable.

• The one subject with questionable adherence (Patient 5) was retained despite failure to meet HRmax goals. This introduces adherence bias.

2. Statistical and Analytical Issues

2.1 Multiple Hypothesis Testing Without Correction

• A wide array of secondary outcomes was tested without adjustment for multiplicity. This raises the likelihood of Type I errors.

• The significant emotional well-being improvement (p < 0.05) could easily be a false positive. Use FDR or Bonferroni adjustment, or qualify results as exploratory.

2.2 Overinterpretation of Non-Significant Trends

• Several non-significant findings (e.g., liver fat reduction of -2.03%, p = 0.22) are framed too optimistically.

• Statements like “trend toward benefit” lack statistical support and should be removed or reworded to reflect the absence of evidence.

2.3 Use of Confidence Intervals

• Many CIs are extremely wide (e.g., insulin AUC: -22101 to 13980), indicating poor precision. This should be acknowledged explicitly in limitations.

3. Issues in Study Design and Execution

3.1 Lack of Blinding in Outcome Assessments

• Apart from MRI fat fraction, all secondary outcomes (e.g., VO2max, SF-36, BP) were unblinded. This introduces measurement bias and threatens validity.

3.2 Heterogeneous Baseline Fat Fraction

• Wide variability in baseline liver fat across groups (10.4% vs. 5.1%) suggests imbalance post-randomization. No stratified randomization or covariate adjustment was applied.

3.3 Missing Pre-Specified Outcomes

• The study originally planned liver biopsies for histological analysis but dropped this due to non-consent. While practical, this change significantly weakens the outcome depth, and should be transparently discussed under limitations with more emphasis.

4. Interpretation and Reporting Concerns

4.1 Inadequate Discussion of Null Results

• The discussion does not address the possibility that HIIT may truly have no effect in CHB-associated steatosis — only that the sample was too small.

• The authors do not critically engage with the negative findings and their implications for pathophysiology or clinical translation.

4.2 Generalizability Issues Ignored

• Only 19 of 695 screened patients were enrolled (2.7%). This extreme attrition suggests selection bias and limits external validity.

• Most participants were middle-aged, urban, and likely motivated — the results are not generalizable to broader CHB populations, especially in LMICs.

4.3 SF-36 Emotional Well-being Score Overstated

• The absolute difference of 18 points is statistically significant but may not be clinically meaningful. Authors must discuss this with reference to known MCIDs for SF-36.

5. Minor comments:

5.1 Incomplete Reporting of Supplementary Material

• Supplementary Tables are frequently referenced but were not included in the manuscript provided.

5.2 Typos and Formatting Errors

• Several errors (e.g., "Hospiutal" instead of "Hospital") and inconsistent units (e.g., glucose AUCs, unclear standard deviations).

5.3 Duplicated or Misreferenced Literature

• Some references appear duplicated (e.g., Hallsworth studies), and some are outdated given the 2025 submission date.

Final Comments: Due to multiple critical issues—particularly underpowering, flawed inclusion criteria, lack of dietary control, and weak statistical rigor—this study cannot support its primary conclusions. If revised, it should be framed explicitly as a feasibility or pilot trial with exploratory outcomes only.

[Note: HTML markup is below. Please do not edit.]

[NOTE: If reviewer comments were submitted as an attachment file, they will be attached to this email and accessible via the submission site. Please log into your account, locate the manuscript record, and check for the action link "View Attachments". If this link does not appear, there are no attachment files.]

While revising your submission, please upload your figure files to the Preflight Analysis and Conversion Engine (PACE) digital diagnostic tool, https://pacev2.apexcovantage.com/. PACE helps ensure that figures meet PLOS requirements. To use PACE, you must first register as a user. Registration is free. Then, login and navigate to the UPLOAD tab, where you will find detailed instructions on how to use the tool. If you encounter any issues or have any questions when using PACE, please email PLOS at figures@plos.org. Please note that Supporting Information files do not need this step.

# Author comment: Author response to Decision Letter 1

**DOI:** 10.1371/journal.pone.0351547.r002

19 Jan 2026

Re: Effect of high-intensity interval training in patients with chronic hepatitis B and hepatic steatosis: a randomised controlled trial

To the Academic Editor

Surya Prakash Bhatt, Ph.D

PLOS ONE

My co-authors and I thank you for considering our manuscript for publication in PLOS ONE. We are grateful for the comments from the Academic Editor, which we have followed and revised the manuscript accordingly. Please find enclosed a revised clean version of the manuscript and a version with revisions marked in tracked changes. We hope that the revised manuscript is satisfactory for publication. Thank you again for your interest in publishing our paper in PLOS ONE.

Yours sincerely

Sofie Dikeledi Kold Jespersen, MD, Ph.D

Reply to Academic Editor comments (author reply in blue)

Final Comments: Due to multiple critical issues—particularly underpowering, flawed inclusion criteria, lack of dietary control, and weak statistical rigor—this study cannot support its primary conclusions. If revised, it should be framed explicitly as a feasibility or pilot trial with exploratory outcomes only.

Response: Thank you for this thorough and thoughtful assessment. We acknowledge the editor’s concerns regarding statistical power, inclusion criteria, and the interpretation of the findings, and we have revised the manuscript to ensure a more cautious interpretation of the findings.

Importantly, the study was designed, prospectively registered, and accompanied by a published protocol as a randomised controlled trial (PMID: 37312098 and ClinicalTrial.gov registration number NCT05265026), and not as a pilot or feasibility study. The original intent was therefore to conduct a definitive RCT. However, due to slower-than-anticipated recruitment, the trial was prematurely discontinued, resulting in a smaller final sample size and limited statistical power.

We fully agree that this limitation must result in a cautious interpretation of the results. Accordingly, we have explicitly stated that the trial was discontinued early due to recruitment challenges and that all analyses should be regarded as exploratory and hypothesis-generating rather than confirmatory. This clarification has been added to the Abstract, Discussion, and Conclusions.

While the reduced sample size precludes definitive conclusions regarding efficacy, we believe that transparent reporting of the trial as conducted—without retrospectively reclassifying it as a pilot study—represents best practice and allows the findings to be interpreted appropriately within their methodological constraints.

We hope that the following revisions will allow the manuscript to be considered for publication.

1.Major Methodological Flaws

1.1 Inadequate Sample Size & Power

• The study was powered for 28 subjects but included only 14 completers. The under-recruitment renders the study severely underpowered, especially for detecting small-to-moderate effects in liver fat fraction.

• Given the non-significant p-values and wide confidence intervals, any inference about effectiveness is unjustified. The conclusion should be reframed as exploratory.

Response: We acknowledge this important limitation and agree with the editor’s assessment. The reduced sample size indeed limited the statistical power of our study, particularly for detecting small-to-moderate effects in liver fat fraction. We have revised the manuscript to explicitly frame the findings as exploratory and rephrased the conclusions to reflect the underpowered nature of the study.

Change in manuscript:

Abstract:

Background & Aims:

Having both chronic hepatitis B and hepatic steatosis increases the risk of liver-related morbidity and mortality. We aimed to investigate if high-intensity interval training could decrease the liver fat-fraction and improve liver status, body composition, lipid- and glucose metabolism, and blood pressure in patients with chronic hepatitis B and hepatic steatosis.

Methods

In a randomised, controlled trial, patients with chronic hepatitis B and hepatic steatosis were randomised 1:1 to high-intensity interval training over 12 weeks or no intervention. The primary outcome was reduction in liver fat-fraction (≥ 2.8 %), assessed by magnetic resonance imaging. Secondary outcomes were body composition, indices of glucose metabolism, blood lipids, blood pressure, alanine aminotransferase, physical fitness assessed by maximal oxygen consumption (VO2max), and self-assessed health. The trial was discontinued before reaching the planned sample size due to slow recruitment.

Results

Nineteen patients were included, and 14 completed the trial (seven per group). Changes in liver fat-fraction showed a between-group difference of -2.03 % [95 % CI: -5.5 to 1.4; p=0.22]. Exercising patients improved VO2max by 5.6 mL/kg/min [1.7 to 9.5; p<0.05] and emotional well-being by 18.07 points [6.6 to 29.6; p<0.05]. The exercise intervention did not affect total body fat -0.35 % [-2.8 to 2.1] or lean body mass 0.6 kg [-0.7 to 1.9]. Changes in glucose tolerance, insulin secretion, Matsuda index -0.89 [-2.5 to 0.5; p=0.18], cholesterol 0.29 mmol/L [-0.3 to 0.9; p=0.32] and systolic blood pressure 6.36 mmHg [-6.4 to 19.1; p=0.29] did not differ significantly between groups.

Conclusions

We did not observe a significant effect of high-intensity interval training on liver fat-fraction in patients with chronic hepatitis B and hepatic steatosis. Due to premature discontinuation and the resulting limited sample size, the study was underpowered, and the findings should be interpreted as exploratory. Nevertheless, the intervention improved physical fitness and emotional well-being.

“Conclusion

This study, the first to investigate the effect of high-intensity interval training on hepatic steatosis in patients with CHB, is inconclusive due to premature discontinuation and the resulting smaller-than-planned sample size, leading to insufficient statistical power. Consequently, no definitive conclusions can be drawn regarding the effect of HIIT on the primary endpoint on liver fat-fraction, and on secondary outcomes, such as body composition, lipid and glucose metabolism, and blood pressure in patients with CHB. The findings should therefore be interpreted as exploratory. However, we found that the intervention was well-tolerated, with no safety concerns identified, and improved cardiorespiratory fitness by improving VO2max and self-reported emotional well-being.“

1.2 Poor Screening Specificity

• CAP >250 dB/m was used to define hepatic steatosis; however, 42% of patients had <5% fat fraction on MRI-PDFF, the actual primary outcome metric. This undermines internal validity.

• The authors themselves suggest >300 dB/m may be more appropriate — this should have been anticipated or at least triggered a mid-study protocol amendment.

Response: We appreciate the editor’s observation. The CAP threshold (>250 dB/m) was specified in the original study protocol based on the best available evidence at the time of study design, most notably the meta-analysis by Karlas et al. (PMID: 28039099), which identified a cut-off around 246 dB/m for the detection of hepatic steatosis. At the time the study was initiated and conducted, there was no established consensus supporting a higher CAP threshold (e.g., >300 dB/m), nor was such a cut-off robustly validated against MRI-PDFF as a reference standard.

Importantly, MRI-PDFF—the primary outcome—was obtained only at study completion for logistical and methodological reasons. Consequently, the observed discrepancy between CAP-based inclusion criteria and MRI-PDFF–defined steatosis could not be identified during the conduct of the study and therefore could not reasonably trigger a protocol amendment or ethical addendum during enrollment.

In retrospect, and based on the present findings, a higher CAP threshold may indeed provide improved specificity when MRI-PDFF is used as the reference standard. This limitation has been explicitly acknowledged in the manuscript, and we agree that future studies should consider higher CAP cut-offs or incorporate baseline MRI-PDFF measurements to better align inclusion criteria with the primary outcome.

Change in manuscript: (under Strengths and Limitations)

“In retrospect, the present results suggest that a higher CAP threshold (e.g., >300 dB/m) may provide improved specificity when MRI-PDFF is used as the reference standard. Future studies should consider using the higher suggested CAP cut-offs or incorporating baseline MRI-PDFF measurements to better align eligibility criteria with the primary outcome. Future studies should consider applying higher CAP cut-offs or combining CAP with baseline MRI-PDFF to better align inclusion criteria with the primary outcome.“

1.3 Absence of Diet Control

• No dietary monitoring or standardization is a critical omission in a metabolic intervention study.

• Diet is a major confounder in hepatic steatosis studies, and the authors admit that at least two participants had major dietary changes.

Response: We acknowledge the editor’s concern and agree that dietary monitoring would have strengthened the study. However, our intention was to isolate the effects of the exercise intervention without imposing dietary restrictions, thereby reflecting real-world conditions. The two cases of major dietary changes were unforeseen and resulted from illness and lifestyle decisions, despite our requirement that participants maintain stable dietary habits throughout the study. This issue has been identified as an important limitation and has been emphasized in the revised manuscript, including that future studies should incorporate dietary monitoring.

Change in manuscript: (under Strengths and Limitations)

“Another limitation is the lack of systematic diet control and monitoring during the intervention. A change in diet has been shown to affect the liver fat-fraction and visceral adiposity greatly (38). Although participants were instructed to maintain stable dietary habits throughout the study at least two patients in this study had major changes in food intake, which were not systematically or quantitively registered. These unanticipated changes, related to illness and religious lifestyle (Ramadan), may have influenced the primary and secondary outcomes and thereby reduced the ability to attribute observed effects solely to the exercise intervention. While the study was designed to examine the effects of exercise under real-world conditions without dietary restrictions, future studies would benefit from incorporating dietary monitoring or control to minimize confounding and improve interpretability.”

1.4 Per-Protocol Bias and ITT Limitations

• Although per-protocol and ITT analyses are reported, the small sample size and lack of blinding make per-protocol results unreliable.

• The one subject with questionable adherence (Patient 5) was retained despite failure to meet HRmax goals. This introduces adherence bias.

Response: We appreciate the editor’s point. While the primary outcome was indeed assessed in a blinded manner, we acknowledge that secondary outcomes were not blinded, which may limit the reliability of those results. In addition, the small sample size and inclusion of Patient 5, who did not consistently achieve the prescribed HRmax target, may have introduced adherence-related bias. In the revised discussion, these limitations are now more clearly described.

Change in manuscript: (under Strengths and Limitations)

“Although both intention-to-treat and per-protocol analyses were performed, the small sample size limits their robustness, and the per-protocol findings in particular should be interpreted with caution. While the primary outcome was assessed in a blinded manner, secondary outcomes were not blinded, which may have introduced measurement bias. In addition, one participant demonstrated suboptimal adherence to the prescribed training intensity yet was retained in the analyses for transparency, which may have contributed to adherence-related bias. Together, these factors further limit the interpretability and generalizability of the findings.”

2. Statistical and Analytical Issues

2.1 Multiple Hypothesis Testing Without Correction

• A wide array of secondary outcomes was tested without adjustment for multiplicity. This raises the likelihood of Type I errors.

• The significant emotional well-being improvement (p < 0.05) could easily be a false positive. Use FDR or Bonferroni adjustment, or qualify results as exploratory.

Response: We agree with the editor’s comment regarding the risk of inflated Type I error due to multiple testing of secondary outcomes. In the revised manuscript, p-values for all secondary outcomes have been adjusted using Bonferroni correction. The effects observed for Emotional well-being and Improvement of cardiorespiratory fitness remain statistically significant after adjustment. All results presented without correction are now explicitly described as exploratory and hypothesis-generating.

2.2 Overinterpretation of Non-Significant Trends

• Several non-significant findings (e.g., liver fat reduction of -2.03%, p = 0.22) are framed too optimistically.

• Statements like “trend toward benefit” lack statistical support and should be removed or reworded to reflect the absence of evidence.

Response: We acknowledge this concern and agree that non-significant findings should not be overstated. The discussion and conclusion sections have been revised to ensure the interpretation is appropriately cautious regarding non-significant trends.

2.3 Use of Confidence Intervals

• Many CIs are extremely wide (e.g., insulin AUC: -22101 to 13980), indicating poor precision. This should be acknowledged explicitly in limitations.

Response. We agree with the editor’s observation. We have explicitly acknowledged this limitation in the revised manuscript.

Change in manuscript: (under Strengths and Limitations)

“This study is primarily limited by a small sample size and substantial heterogeneity among participants with respect to ethnicity, body weight, and liver fat fraction. Together, these factors led to wide confidence intervals, diminished statistical power, and an increased risk of type II error, thereby constraining the robustness of the conclusions.”

3. Issues in Study Design and Execution

3.1 Lack of Blinding in Outcome Assessments

• Apart from MRI fat fraction, all secondary outcomes (e.g., VO2max, SF-36, BP) were unblinded. This introduces measurement bias and threatens validity.

Response: We acknowledge this limitation. While the primary outcome was assessed in a blinded manner, secondary outcomes were not. The Limitations section has been revised to more clearly acknowledge this limitation.

Change in manuscript: (under Strength and Limitations)

“While the primary outcome was assessed in a blinded manner, secondary outcomes were not blinded, which may have introduced measurement bias. In addition, one participant demonstrated suboptimal adherence to the prescribed training intensity yet was retained in the analyses for transparency, which may have contributed to adherence-related bias. Together, these factors further limit the interpretability and generalizability of the findings.”

3.2 Heterogeneous Baseline Fat Fraction

• Wide variability in baseline liver fat across groups (10.4% vs. 5.1%) suggests imbalance post-randomization. No stratified randomization or covariate adjustment was applied.

Response: We acknowledge the observed baseline imbalance in liver fat fraction between groups. This difference likely reflects random variation due to randomization and the small sample size; with the originally planned sample size less variability would have been expected. This limitation is noted in the revised manuscript and clarify its potential impact on the interpretation of results.

Change in manuscript: (under Strength and Limitations)

“Another important limitation of this study concerns the specificity of the screening criterion used to define hepatic steatosis. A CAP threshold >250 dB/m was applied at inclusion; however, 42% of participants had a hepatic fat fraction < 5% when assessed by MRI-PDFF, the primary outcome measure. This mismatch likely reduced the study’s ability to detect intervention-related effects and

Attachment Submitted filename: Response to editor.docx

# Peer review: Decision Letter 1

**DOI:** 10.1371/journal.pone.0351547.r003

**Authors:** Sophia Eugenia Martínez-Vázquez

2 Apr 2026

<div>PONE-D-25-33230R1

Effect of high-intensity interval training in patients with chronic hepatitis B and hepatic steatosis: a randomised controlled trial

PLOS One

Dear Dr. Jespersen,

Thank you for submitting your manuscript to PLOS ONE. After careful consideration, we feel that it has merit but does not fully meet PLOS ONE’s publication criteria as it currently stands. Therefore, we invite you to submit a revised version of the manuscript that addresses the points raised during the review process.

Please submit your revised manuscript by May 17 2026 11:59PM. If you will need more time than this to complete your revisions, please reply to this message or contact the journal office at plosone@plos.org. When you're ready to submit your revision, log on to https://www.editorialmanager.com/pone/ and select the 'Submissions Needing Revision' folder to locate your manuscript file.

Please include the following items when submitting your revised manuscript:

- A letter that responds to each point raised by the academic editor and reviewer(s). You should upload this letter as a separate file labeled 'Response to Reviewers'.
- A marked-up copy of your manuscript that highlights changes made to the original version. You should upload this as a separate file labeled 'Revised Manuscript with Track Changes'.
- An unmarked version of your revised paper without tracked changes. You should upload this as a separate file labeled 'Manuscript'.

If you would like to make changes to your financial disclosure, please include your updated statement in your cover letter. Guidelines for resubmitting your figure files are available below the reviewer comments at the end of this letter.

If applicable, we recommend that you deposit your laboratory protocols in protocols.io to enhance the reproducibility of your results. Protocols.io assigns your protocol its own identifier (DOI) so that it can be cited independently in the future. For instructions see: https://journals.plos.org/plosone/s/submission-guidelines#loc-laboratory-protocols. Additionally, PLOS ONE offers an option for publishing peer-reviewed Lab Protocol articles, which describe protocols hosted on protocols.io. Read more information on sharing protocols at https://plos.org/protocols?utm_medium=editorial-email&utm_source=authorletters&utm_campaign=protocols.

As the corresponding author, your ORCID iD is verified in the submission system and will appear in the published article. PLOS supports the use of ORCID, and we encourage all coauthors to register for an ORCID iD and use it as well. Please encourage your coauthors to verify their ORCID iD within the submission system before final acceptance, as unverified ORCID iDs will not appear in the published article. *Only*   the individual author can complete the verification step; PLOS staff *cannot*   verify ORCID iDs on behalf of authors.

We look forward to receiving your revised manuscript.

Kind regards,

Sophia Eugenia Martínez-Vázquez, Ph.D.

Academic Editor

PLOS One

Journal Requirements:

If the reviewer comments include a recommendation to cite specific previously published works, please review and evaluate these publications to determine whether they are relevant and should be cited. There is no requirement to cite these works unless the editor has indicated otherwise.

Additional Editor Comments:

Dear Authors

Thank you for the opportunity to revise your manuscript about "Effect of high-intensity interval training in patients with chronic hepatitis B and hepatic steatosis: a randomised controlled trial". Some points called my attention:

1. Please explain the real difference of hepatic steatosis by chronic hepatitis B in terms of treatment, that is to say, why a clinical study was needed to demonstrate that the same recommendation of exercise for other origin of the liver steatosis need to be proved in CHB?

2. About sample size, please state if it was not reached because the prevalence of the disease in your hospital, city or country; or other condition.

3. Explain the reason that the initial elastography was not in fasting. It generates concerns about the real steatosis stage at the time of diagnosis and then in the change of stage during the study.

[Note: HTML markup is below. Please do not edit.]

Reviewers' comments:

Reviewer's Responses to Questions

**Comments to the Author**

1. If the authors have adequately addressed your comments raised in a previous round of review and you feel that this manuscript is now acceptable for publication, you may indicate that here to bypass the “Comments to the Author” section, enter your conflict of interest statement in the “Confidential to Editor” section, and submit your "Accept" recommendation.

Reviewer #1: (No Response)

Reviewer #2: (No Response)

**********

2. Is the manuscript technically sound, and do the data support the conclusions?

The manuscript must describe a technically sound piece of scientific research with data that supports the conclusions. Experiments must have been conducted rigorously, with appropriate controls, replication, and sample sizes. The conclusions must be drawn appropriately based on the data presented.

Reviewer #1: Partly

Reviewer #2: Partly

**********

3. Has the statistical analysis been performed appropriately and rigorously?

Reviewer #1: Yes

Reviewer #2: Yes

**********

4. Have the authors made all data underlying the findings in their manuscript fully available?

The PLOS Data policy requires authors to make all data underlying the findings described in their manuscript fully available without restriction, with rare exception (please refer to the Data Availability Statement in the manuscript PDF file). The data should be provided as part of the manuscript or its supporting information, or deposited to a public repository. For example, in addition to summary statistics, the data points behind means, medians and variance measures should be available. If there are restrictions on publicly sharing data—e.g. participant privacy or use of data from a third party—those must be specified.

Reviewer #1: Yes

Reviewer #2: Yes

**********

5. Is the manuscript presented in an intelligible fashion and written in standard English?

PLOS ONE does not copyedit accepted manuscripts, so the language in submitted articles must be clear, correct, and unambiguous. Any typographical or grammatical errors should be corrected at revision, so please note any specific errors here.

Reviewer #1: Yes

Reviewer #2: Yes

**********

6. Review Comments to the Author

Please use the space provided to explain your answers to the questions above. You may also include additional comments for the author, including concerns about dual publication, research ethics, or publication ethics. (Please upload your review as an attachment if it exceeds 20,000 characters)

Reviewer #1: GENERAL COMMENTS

Thank you for the opportunity to review manuscript PONE-D-25-33230R1 titled “Effect of high-intensity interval training in patients with chronic hepatitis B and hepatic

steatosis: a randomised controlled trial”. I note here that I have not previously reviewed this.

Despite the need to end this study before a sufficient sample size was reached, I think this is a timely, important, objective, and well-done study overall, The former notwithstanding, two broad concerns, included in my specific comments below, have to do with whether some of the participants were already exercising prior to enrollment as well as whether the investigative team accounted for dropouts when determining sample size requirements. Finally, some minor improvement in the writing of this manuscript in the English-language is needed.

SPECIFIC COMMENTS

*Page 3 and 4 (Abstract) – A nicely done, honest, and objective abstract.

*Page 5, lines 109 through 119 (Materials and Methods, Participants and eligibility) – Did you not include as part of your inclusion criteria that participants were not currently engaged in a regular exercise program?

*Page 6, lines 122 through 128 (Materials and Methods, Intervention) – Please tell the reader if these exercise sessions were supervised, and if so, by who. As I read ahead to page 22, line 389, I see that the exercise sessions were supervised.

*Page 6, lines 132 through 142 (Materials and Methods, Outcomes, Liver fat-fraction) – Please provide data on the accuracy and precision of your MRI proton density fat-fraction assessment. The same for the assessment of your other outcomes, which in some cases would be validity and reliability data, e.g., SF-36.

*Page 9, line 194 through 199 (Materials and Methods, Randomization and blinding) – First, please tell the reader who was responsible for conducting the randomization. Second, rather than saying “a co-author” on line 196, state the author per the author order in the manuscript, for example, “The second author assessed…”

*Page 10, lines 202 through 204 (Materials and Methods, Changes from the original protocol) – Unless part of the original protocol, it would seem appropriate to tell the reader that you stopped the trial early along with why the trial was stopped early.

*Page 10, lines 215 through 218 (Materials and Methods, Sample size considerations) – In your protocol, was any plan made to account for dropouts when determining your initial sample size? If so, then please describe. If not, then please state that also. As we all know, dropouts in exercise studies are very common, especially for those with chronic conditions.

*Pages 10 and 11, lines 221 through 241 (Materials and Methods, Statistical analysis) – First, for your baseline characteristics (lines 221 through 223), suggest that you test for between-group differences and tell the reader what test(s) were used. Second, on lines 232 and 233, there are multiple different ways to conduct intention-to-treat analysis. Please describe the one you used, for example, last observation carried forward.

*Pages 20 through 24, lines 336 through 450 (Discussion) – For ease of reading, suggest you partition your Discussion into the following subsections and address: (1) Overall findings, (2) Implications for research, (3) Implications for practice, (4) Strengths and potential limitations. Also, while a secondary outcome in your study, it may be worth mentioning, with a citation or two, that VO2 max is an important clinical marker, regardless of the population studied.

*Pages 12 through 14 (Table 1) – As previously suggested, analyze for statistically significant differences in baseline characteristics between the exercise and control groups. This could be done by simply adding a column with a p-value.

END OF REVIEW

Reviewer #2: General comments:

The reframing of the findings and the correction of the text accordingly are extremely welcome. The emphasis on effect size and 95% CI rather than post-hoc power is a correct approach. However, please clarify which estimation is being targeted.

If MRI-PDFF was measured only at the end of the study, the change cannot be calculated. However, group differences can be reported at the endline. This approach risks bias if baseline imbalance exists. If a change has been reported, MRI-PDFF must have been measured at baseline as well. Therefore, the statement 'MRI-PDFF was only obtained at the end of the study' is inconsistent and should be corrected.

While the lack of dietary control could have a strong confounding effect on metabolic endpoints, and this deficiency is appropriately explained in the Limitations section of the text, it remains unclear to what extent the major dietary changes reported in the two participants influenced the findings, and the current response is incomplete.

It is positive from a transparency perspective that you stated PP/ITT analyses were performed and that participants with adhesion problems were excluded from the analyses. However, clarification may be needed regarding how ITT was implemented, the assumption of missing data, and the decision not to use LOCF. Furthermore, the pre-defined adhesion thresholds for PP analysis, how these thresholds are defined in the protocol, and how they are measured should be clarified.

The authors applied a Bonferroni adjustment, and EWB and VO₂max remained statistically significant. For transparency, please specify the number of hypotheses included in the multiplicity adjustment and report, for each secondary endpoint, both the raw p‑values and the Bonferroni‑adjusted p‑values. Also indicate that tests were two‑sided and provide the corresponding α_adj (ex., 0.05/K).

**********

7. PLOS authors have the option to publish the peer review history of their article (what does this mean?). If published, this will include your full peer review and any attached files.

If you choose “no”, your identity will remain anonymous but your review may still be made public.

**Do you want your identity to be public for this peer review?**   For information about this choice, including consent withdrawal, please see our Privacy Policy.

Reviewer #1: No

Reviewer #2: **Yes:**  Assoc. Prof. Senay Akin

**********

[NOTE: If reviewer comments were submitted as an attachment file, they will be attached to this email and accessible via the submission site. Please log into your account, locate the manuscript record, and check for the action link "View Attachments". If this link does not appear, there are no attachment files.]

To ensure your figures meet our technical requirements, please review our figure guidelines: https://journals.plos.org/plosone/s/figures

You may also use PLOS’s free figure tool, NAAS, to help you prepare publication quality figures: https://journals.plos.org/plosone/s/figures#loc-tools-for-figure-preparation.

NAAS will assess whether your figures meet our technical requirements by comparing each figure against our figure specifications.

# Author comment: Author response to Decision Letter 2

**DOI:** 10.1371/journal.pone.0351547.r004

13 May 2026

Effect of high-intensity interval training in patients with chronic hepatitis B and hepatic steatosis: a randomized controlled trial

To the Reviewers and Academic Editor, Sophia Eugenia Martínez-Vázquez, PhD

Dear Reviewers and Academic Editor,

My co-authors and I sincerely thank you for considering and reviewing our manuscript for publication in PLOS ONE. We are very grateful for the thoughtful and constructive comments provided by both Reviewers and the Academic Editor.

We have carefully addressed all comments and revised the manuscript accordingly. Please find enclosed a clean revised version of the manuscript and a version with tracked changes highlighting the revisions. We hope that the revisions adequately address your concerns and that the manuscript is now suitable for publication.

Thank you again for your time and consideration.

Yours sincerely, on behalf of the authors

Sofie Jespersen, MD, PhD

Department of Infectious Diseases

Copenhagen University Hospital, Hvidovre

Denmark

Reply to the Academic Editors Comments:

Dear Authors

Thank you for the opportunity to revise your manuscript about "Effect of high-intensity interval training in patients with chronic hepatitis B and hepatic steatosis: a randomized controlled trial".

Reply: Thank you very much for taking the time to review and comment on our manuscript.

Some points called my attention:

1. Please explain the real difference of hepatic steatosis by chronic hepatitis B in terms of treatment, that is to say, why a clinical study was needed to demonstrate that the same recommendation of exercise for other origin of the liver steatosis need to be proved in CHB?

Reply: We agree that the rationale for conducting a clinical study specifically in patients with CHB and hepatic steatosis could be articulated more clearly. While lifestyle interventions, including exercise, are well established in the management of hepatic steatosis of metabolic origin (MASLD, previously terminology NAFLD, which was used when designing the study) with a recommendation of >150 min/week of moderate or 75 min/week of vigorous intensity activity( ref: European Association for the Study of the Liver (EASL); European Association for the Study of Diabetes (EASD); European Association for the Study of Obesity (EASO). EASL-EASD-EASO Clinical Practice Guidelines on the management of metabolic dysfunction-associated steatotic liver disease (MASLD). J Hepatol. 2024 Sep;81(3):492-542. doi: 10.1016/j.jhep.2024.04.031. Epub 2024 Jun 7. PMID: 38851997.), it cannot be assumed that these effects are directly transferable to patients with CHB.

The underlying pathophysiology of hepatic steatosis in CHB may differ from that of MASLD in several important respects. In CHB, viral factors, host immune response, and interactions between hepatitis B virus replication and hepatic lipid metabolism may influence both the development of steatosis and its response to interventions. Additionally, hepatic steatosis in CHB has been associated with altered disease progression, including potential effects on fibrosis progression and hepatocellular carcinoma risk, which may not mirror patterns seen in MASLD.

Furthermore, current clinical guidelines, including those from EASL concerning management of CHB (ref: European Association for the Study of the Liver. EASL Clinical Practice Guidelines on the management of hepatitis B virus infection. J Hepatol. 2025 Aug;83(2):502-583. doi: 10.1016/j.jhep.2025.03.018. Epub 2025 May 10. PMID: 40348683.), do not provide specific recommendations for managing patients with concomitant CHB and hepatic steatosis. As a result, there is a lack of evidence-based guidance on whether exercise interventions, such as high-intensity interval training, are comparably efficacious and safe in this population.

Therefore, we thought that a clinical study was needed to specifically investigate whether the beneficial effects of exercise observed in MASLD populations could be replicated in patients with CHB, and to better inform clinical management in this distinct and understudied group of patients.

We have clarified this rationale in the revised introduction.

Page 4-5, line 90-100: “Hepatic steatosis in patients with CHB may differ from steatosis of metabolic origin in terms of underlying pathophysiology. In CHB, viral factors and host–virus interactions may influence lipid metabolism and disease progression, raising uncertainty as to whether treatment strategies established for NAFLD can be directly applied to this population.

The European Association for the Study of the Liver (EASL) guidelines provide no guidance on how to manage patients with both CHB and hepatic steatosis (8). Previous similar exercise intervention studies in patients with NAFLD (without CHB) have shown decreased liver fat-fraction and improved peripheral insulin resistance and liver fatty acid metabolism (9–11). Therefore, because patients with CHB have been excluded from these studies, evidence for similar benefits in this group is lacking.”

2. About sample size, please state if it was not reached because the prevalence of the disease in your hospital, city or country; or other condition.

Reply: We believe that another reason for not reaching the target sample size was the relatively low prevalence of the condition in our country compared to other regions such as Asia and Africa, where the burden of disease is substantially higher. This inherently limited the pool of eligible patients available for inclusion. It is estimated that the prevalence of chronic hepatitis B in Denmark is approximately 14.000 persons which corresponds to a 0.3 % of the population (ref. Bollerup S et al. see below)

We have clarified this point in the revised manuscript with the following:

Page 26, line 454 to 457: “Due to slow recruitment, we decided to close the study before reaching the intended number of patients. This challenge is likely multifactorial. CHB has a relatively low prevalence in Denmark (approximately 0.3%) (ref. Bollerup S, Wessman M, Hansen JF, Nielsen S, Hay G, Cowan S, Krarup H, Omland L, Jepsen P, Weis N, Christensen PB. Increasing prevalence of chronic hepatitis B virus infection and low linkage to care in Denmark on 31 December 2016 - an update based on nationwide registers. Infect Dis (Lond). 2023 Jan;55(1):17-26. doi: 10.1080/23744235.2022.2125065. Epub 2022 Oct 11. PMID: 36221255.). ), which restricts the number of eligible patients available for inclusion compared to higher-prevalence regions. In addition, patient-related factors may have influenced recruitment.

3. Explain the reason that the initial elastography was not in fasting. It generates concerns about the real steatosis stage at the time of diagnosis and then in the change of stage during the study.

Reply: We agree that fasting conditions may influence elastography measurements and, consequently, the assessment of steatosis stage.

In our clinical setting, it was not feasible to ensure that all patients were fasting prior to the baseline elastography assessment, as participants were recruited during routine outpatient visits. However, all patients are regularly followed in the clinic and are advised to attend consultations in a fasting state, as elastography is performed routinely as part of their clinical care.

Importantly, this limitation applied uniformly at baseline and reflects real-world clinical practice. Nonetheless, we acknowledge that non-fasting status may have introduced variability in TE-based steatosis measurements at baseline, and we hope to have addressed this transparently in the manuscript.

We would also like to emphasize that MRI-PDFF, for which all patients were examined under fasting conditions, was the primary outcome measure for hepatic steatosis in this study. TE was used as part of the initial assessment; however, in retrospect, a stricter standardization of fasting conditions for baseline TE may have been preferable.

Here is the revision we made in the Methods section:

Page 8, line 162-164: “TE performed prior to inclusion was not consistently conducted under fasting conditions, however intended, as these assessments were carried out during routine outpatient visits where fasting could not be systematically ensured. “

Reply to reviewers' comments:

Reviewer #1: GENERAL COMMENTS

Thank you for the opportunity to review manuscript PONE-D-25-33230R1 titled “Effect of high-intensity interval training in patients with chronic hepatitis B and hepatic

steatosis: a randomised controlled trial”. I note here that I have not previously reviewed this.

Despite the need to end this study before a sufficient sample size was reached, I think this is a timely, important, objective, and well-done study overall, The former notwithstanding, two broad concerns, included in my specific comments below, have to do with whether some of the participants were already exercising prior to enrollment as well as whether the investigative team accounted for dropouts when determining sample size requirements. Finally, some minor improvement in the writing of this manuscript in the English-language is needed.

Reply: Thank you very much for your positive and encouraging feedback. We appreciate your constructive comments, and we have addressed each point in the revised manuscript.

SPECIFIC COMMENTS

*Page 3 and 4 (Abstract) – A nicely done, honest, and objective abstract.

Reply: Thank you.

*Page 5, lines 109 through 119 (Materials and Methods, Participants and eligibility) – Did you not include as part of your inclusion criteria that participants were not currently engaged in a regular exercise program?

Reply: At inclusion, all participants were asked about their exercise habits, and none were engaged in regular high-intensity exercise. Some participants did have access to a gym, but they were not performing structured high-intensity exercise or attending on a weekly basis. Formally, being currently engaged in a regular exercise program was not an exclusion criterion; in retrospect, incorporating this criterion might have been preferable to further standardize baseline physical activity levels.

*Page 6, lines 122 through 128 (Materials and Methods, Intervention) – Please tell the reader if these exercise sessions were supervised, and if so, by who. As I read ahead to page 22, line 389, I see that the exercise sessions were supervised.

Reply: We have clarified in the revised manuscript that all exercise sessions were supervised. Specifically, we have added the following text to the Methods section:

Page 6, line 134-136: “The supervision of each exercise session was performed by personnel from the Centre of Physical Activity Research, including sports science students, physiotherapy students, and medical students.”

For additional detail on the intervention, we also refer to our previously published protocol, which provides a comprehensive description of the exercise program and its supervision. (ref: Jespersen S, Plomgaard P, Madsbad S, Hansen AE, Bandholm T, Pedersen BK, et al. Effect of aerobic exercise training on the fat fraction of the liver in persons with chronic hepatitis B and hepatic steatosis: Trial protocol for a randomized controlled intervention trial- The FitLiver study. Trials. 2023 Jun 13;24(1):398)

*Page 6, lines 132 through 142 (Materials and Methods, Outcomes, Liver fat-fraction) – Please provide data on the accuracy and precision of your MRI proton density fat-fraction assessment. The same for the assessment of your other outcomes, which in some cases would be validity and reliability data, e.g., SF-36.

Reply: We have clarified the accuracy and reliability of the outcome measures in the Methods section and added appropriate references.

Page 7, line 152-156: “MRI-PDFF is a well-validated, non-invasive method for quantifying liver fat, demonstrating high diagnostic accuracy and reproducibility. Meta-analytic data show excellent performance across steatosis grades, with areas under the curve (AUC) values ranging from 0.91 to 0.97 and high sensitivity and specificity, supporting its robustness for both detection and grading of hepatic steatosis . (ref: Azizi N, Naghibi H, Shakiba M, Morsali M, Zarei D, Abbastabar H, Ghanaati H. Evaluation of MRI proton density fat fraction in hepatic steatosis: a systematic review and meta-analysis. Eur Radiol. 2025 Apr;35(4):1794-1807. doi: 10.1007/s00330-024-11001-1. Epub 2024 Sep 10. PMID: 39254718.)”

And

Page 10, line 206-211: ”SF-36 is a well-validated and reliable instrument for assessing health-related quality of life (ref: Brazier JE, Harper R, Jones NM, O'Cathain A, Thomas KJ, Usherwood T, Westlake L. Validating the SF-36 health survey questionnaire: new outcome measure for primary care. BMJ. 1992 Jul 18;305(6846):160-4. doi: 10.1136/bmj.305.6846.160. PMID: 1285753; PMCID: PMC1883187.). The SF-36 has consistently demonstrated strong internal consistency, with Cronbach’s alpha coefficients typically exceeding 0.85, and good test–retest reliability, with intraclass correlation coefficients often above 0.70. Its validity and responsiveness to changes in health status have been well documented across diverse populations. (ref: Kwan YH, Fong WW, Lui NL, Yong ST, Cheung YB, Malhotra R, Østbye T, Thumboo J. Validity and reliability of the Short Form 36 Health Surveys (SF-36) among patients with spondyloarthritis in Singapore. Rheumatol Int. 2016 Dec;36(12):1759-1765. doi: 10.1007/s00296-016-3567-3. Epub 2016 Sep 23. PMID: 27664139. And Wu Q, Chen Y, Zhou Y, Zhang X, Huang Y, Liu R. Reliability, validity, and sensitivity of short-form 36 health survey (SF-36) in patients with sick sinus syndrome. Medicine (Baltimore). 2023 Jun 16;102(24):e33979. doi: 10.1097/MD.0000000000033979. PMID: 37327281; PMCID: PMC10270486.)”

*Page 9, line 194 through 199 (Materials and Methods, Randomization and blinding) – First, please tell the reader who was responsible for conducting the randomization. Second, rather than saying “a co-author” on line 196, state the author per the author order in the manuscript, for example, “The second author assessed…”

Reply: We have clarified the randomization process in the revised manuscript, specifying who was responsible and using author order rather than a general description under the section Randomization and blinding.

Page 10, line 215-223: “The randomization sequence was generated using Sealedenvelope.com with varying block sizes, as described in detail elsewhere (Jespersen S, Plomgaard P, Madsbad S, Hansen AE, Bandholm T, Pedersen BK, Ritz C, Weis N, Krogh-Madsen R. Effect of aerobic exercise training on the fat fraction of the liver in persons with chronic hepatitis B and hepatic steatosis: Trial protocol for a randomized controlled intervention trial- The FitLiver study. Trials. 2023 Jun 13;24(1):398. doi: 10.1186/s13063-023-07385-y. PMID: 37312098; PMCID: PMC10262472). The envelopes were prepared by an independent researcher not involved in participant enrollment or assessment. The first author (S. Jespersen) enrolled participants and opened the envelopes sequentially immediately after baseline assessments, assigning participants irreversibly to their group. Neither the patients nor the primary investigator were blinded. The third author (C.Durrer) assessed the primary outcome of liver fat-fraction blinded without prior involvement with participants or examinations, and the primary investigator assessed the secondary outcomes unblinded.“

*Page 10, lines 202 through 204 (Materials and Methods, Changes from the original protocol) – Unless part of the original protocol, it would seem appropriate to tell the reader that you stopped the trial early along with why the trial was stopped early.

Reply: Very good point. We have added the following sentence to the “Changes from the original protocol” section:

Page 11, line 228-229: “Additionally, the trial was stopped early before reaching the planned sample size due to slow recruitment.”

*Page 10, lines 215 through 218 (Materials and Methods, Sample size considerations) – In your protocol, was any plan made to account for dropouts when determining your initial sample size? If so, then please describe. If not, then please state that also. As we all know, dropouts in

Attachment Submitted filename: Response to reviewers 13052026.docx

# Peer review: Decision Letter 2

**DOI:** 10.1371/journal.pone.0351547.r005

**Authors:** Sophia Eugenia Martínez-Vázquez

28 May 2026

Effect of high-intensity interval training in patients with chronic hepatitis B and hepatic steatosis: a randomised controlled trial

PONE-D-25-33230R2

Dear Dr. Jespersen,

We’re pleased to inform you that your manuscript has been judged scientifically suitable for publication and will be formally accepted for publication once it meets all outstanding technical requirements.

Within one week, you’ll receive an e-mail detailing the required amendments. When these have been addressed, you’ll receive a formal acceptance letter and your manuscript will be scheduled for publication.

An invoice will be generated when your article is formally accepted. Please note, if your institution has a publishing partnership with PLOS and your article meets the relevant criteria, all or part of your publication costs will be covered. Please make sure your user information is up-to-date by logging into Editorial Manager at Editorial Manager® and clicking the ‘Update My Information' link at the top of the page. For questions related to billing, please contact billing support.

If your institution or institutions have a press office, please notify them about your upcoming paper to help maximize its impact. If they’ll be preparing press materials, please inform our press team as soon as possible -- no later than 48 hours after receiving the formal acceptance. Your manuscript will remain under strict press embargo until 2 pm Eastern Time on the date of publication. For more information, please contact onepress@plos.org.

Kind regards,

Sophia Eugenia Martínez-Vázquez, Ph.D.

Academic Editor

PLOS One

Additional Editor Comments (optional):

Dear authors,

Thank you for your answers, I just have to express my congratulations for this effort.

Reviewers' comments:

Reviewer's Responses to Questions

**Comments to the Author**

1. If the authors have adequately addressed your comments raised in a previous round of review and you feel that this manuscript is now acceptable for publication, you may indicate that here to bypass the “Comments to the Author” section, enter your conflict of interest statement in the “Confidential to Editor” section, and submit your "Accept" recommendation.

Reviewer #1: All comments have been addressed

**********

2. Is the manuscript technically sound, and do the data support the conclusions?

The manuscript must describe a technically sound piece of scientific research with data that supports the conclusions. Experiments must have been conducted rigorously, with appropriate controls, replication, and sample sizes. The conclusions must be drawn appropriately based on the data presented.

Reviewer #1: Yes

**********

3. Has the statistical analysis been performed appropriately and rigorously?

Reviewer #1: Yes

**********

4. Have the authors made all data underlying the findings in their manuscript fully available?

The PLOS Data policy requires authors to make all data underlying the findings described in their manuscript fully available without restriction, with rare exception (please refer to the Data Availability Statement in the manuscript PDF file). The data should be provided as part of the manuscript or its supporting information, or deposited to a public repository. For example, in addition to summary statistics, the data points behind means, medians and variance measures should be available. If there are restrictions on publicly sharing data—e.g. participant privacy or use of data from a third party—those must be specified.

Reviewer #1: Yes

**********

5. Is the manuscript presented in an intelligible fashion and written in standard English?

PLOS ONE does not copyedit accepted manuscripts, so the language in submitted articles must be clear, correct, and unambiguous. Any typographical or grammatical errors should be corrected at revision, so please note any specific errors here.

Reviewer #1: Yes

**********

6. Review Comments to the Author

Please use the space provided to explain your answers to the questions above. You may also include additional comments for the author, including concerns about dual publication, research ethics, or publication ethics. (Please upload your review as an attachment if it exceeds 20,000 characters)

Reviewer #1: GENERAL COMMENTS

Thank you for the opportunity to review revised manuscript PONE-D-25-33230R2 titled “Effect of high-intensity interval training in patients with chronic hepatitis B and hepatic

steatosis: a randomised controlled trial”.

In my opinion, the authors have done an excellent job in responding to my previous comments and suggestions. I have no further comments or suggestions for improvement.

END OF REVIEW

**********

7. PLOS authors have the option to publish the peer review history of their article (what does this mean?). If published, this will include your full peer review and any attached files.

If you choose “no”, your identity will remain anonymous but your review may still be made public.

**Do you want your identity to be public for this peer review?**   For information about this choice, including consent withdrawal, please see our Privacy Policy.

Reviewer #1: No

**********

# Editor report: Acceptance letter

**DOI:** 10.1371/journal.pone.0351547.r006

**Authors:** Sophia Eugenia Martínez-Vázquez

PONE-D-25-33230R2

PLOS One

Dear Dr. Jespersen,

I'm pleased to inform you that your manuscript has been deemed suitable for publication in PLOS One. Congratulations! Your manuscript is now being handed over to our production team.

At this stage, our production department will prepare your paper for publication. This includes ensuring the following:

* All references, tables, and figures are properly cited

* All relevant supporting information is included in the manuscript submission,

* There are no issues that prevent the paper from being properly typeset

You will receive further instructions from the production team, including instructions on how to review your proof when it is ready. Please keep in mind that we are working through a large volume of accepted articles, so please give us a few days to review your paper and let you know the next and final steps.

Lastly, if your institution or institutions have a press office, please let them know about your upcoming paper now to help maximize its impact. If they'll be preparing press materials, please inform our press team within the next 48 hours. Your manuscript will remain under strict press embargo until 2 pm Eastern Time on the date of publication. For more information, please contact onepress@plos.org.

You will receive an invoice from PLOS for your publication fee after your manuscript has reached the completed accept phase. If you receive an email requesting payment before acceptance or for any other service, this may be a phishing scheme. Learn how to identify phishing emails and protect your accounts at https://explore.plos.org/phishing.

If we can help with anything else, please email us at customercare@plos.org.

Thank you for submitting your work to PLOS ONE and supporting open access.

Kind regards,

PLOS ONE Editorial Office Staff

on behalf of

Dr. Sophia Eugenia Martínez-Vázquez

Academic Editor

PLOS One
